# Beyond plant defense: insights on the potential of salicylic and methylsalicylic acid to contain growth of the phytopathogen *Botrytis cinerea*

**DOI:** 10.3389/fpls.2015.00859

**Published:** 2015-10-16

**Authors:** Cindy Dieryckx, Vanessa Gaudin, Jean-William Dupuy, Marc Bonneu, Vincent Girard, Dominique Job

**Affiliations:** ^1^Laboratoire Mixte UMR 5240, Plateforme de Protéomique, Centre National de la Recherche ScientifiqueLyon, France; ^2^Plateforme Protéome, Centre de Génomique Fonctionnelle, Université de BordeauxBordeaux, France

**Keywords:** salicylic acid, *Botrytis cinerea*, fungal growth, proteomics, secretomics

## Abstract

Using *Botrytis cinerea* we confirmed in the present work several previous studies showing that salicylic acid, a main plant hormone, inhibits fungal growth *in vitro*. Such an inhibitory effect was also observed for the two salicylic acid derivatives, methylsalicylic and acetylsalicylic acid. In marked contrast, 5-sulfosalicylic acid was totally inactive. Comparative proteomics from treated vs. control mycelia showed that both the intracellular and extracellular proteomes were affected in the presence of salicylic acid or methylsalicylic acid. These data suggest several mechanisms that could potentially account for the observed fungal growth inhibition, notably pH regulation, metal homeostasis, mitochondrial respiration, ROS accumulation and cell wall remodeling. The present observations support a role played by the phytohormone SA and derivatives in directly containing the pathogen. Data are available via ProteomeXchange with identifier PXD002873.

## Introduction

Filamentous fungi are the major plant pathogens that cause multi-millions of US dollars in pre- and post-harvest crop losses worldwide (Bolton et al., 2006). In particular *Botrytis cinerea* (*Botrytis*), a necrotrophic and polyphagous fungus, is able to infect over 200 plants corresponding mostly to flowering plants of temperate and subtropical regions (Mansfield, 1980; Elad, 1997; Williamson et al., 2007). The availability of molecular tools has considerably advanced our understanding of the infection strategies of this fungus (Hahn et al., 2014). Furthermore, its genome has been sequenced revealing over 16,000 protein-coding genes (Amselem et al., 2011; Staats and van Kan, 2012; Hahn et al., 2014). Hence, *Botrytis* is now a widely used fungal model, being among the top 10 fungal pathogens in molecular plant pathology (Dean et al., 2012), thus allowing to unravel genes accounting for pathogenicity (Amselem et al., 2011; Aguileta et al., 2012; Dean et al., 2012; Heard et al., 2015) and for the development of fungicides with novel modes of action (Tietjen et al., 2005).

Infection by a phytopathogenic fungus can only occur if the pathogen possesses all the necessary molecules to override plant defenses (van Baarlen et al., 2007; Hahn et al., 2014). Indeed, during the infection process the plant has the potential to mount a very effective defense for killing/confining its aggressor. In this process, the plant hormone salicylic acid (SA) is a key signal in the induction of the plant immune response to pathogens, and is therefore of great interest in plant pathology and crop protection. This hormone is responsible for controlling critical aspects of both basal and resistance gene based immunity, and for promotion of the long lasting, broadly effective immunity termed systemic acquired resistance (SAR) (Gaffney et al., 1993; Vlot et al., 2009; An and Mou, 2011). Such SAR enables plants to prepare for another attack and defend themselves more effectively against the pathogen (Dangl and Jones, 2001; Durrant and Dong, 2004). A late response is then implemented through the production of defense proteins and phytoalexins and the strengthening of the plant cell wall (Williamson et al., 2007; Mengiste, 2012; Hahn et al., 2014). Besides this function during biotic stress, it has also been found that SA plays a role in the plant response to abiotic stresses such as drought, chilling, heavy metal toxicity, heat, and osmotic stress as well as during plant growth and development (reviewed by Rivas-San Vicente and Plasencia, 2011).

For more than 200 years, SA (2-hydroxy benzoic acid) and derivatives have been studied for their medicinal use in humans (Vane and Botting, 2003; Jones, 2011). However, the extensive signaling role of SA in plants, particularly in defense against pathogens, has only become evident during the past 20 years (Ferrari et al., 2003; Rajjou et al., 2006; van Loon et al., 2006; Vlot et al., 2009; Zipfel, 2009; Hayat et al., 2010; El Oirdi et al., 2011; Caarls et al., 2015). SA derivatives are also widely distributed in plants. Methylsalicylate (MeSA; methyl 2-hydroxybenzoate) deserves special attention, as it is a volatile long distance signaling molecule that moves from infected to the non-infected tissues through phloem (Shulaev et al., 1997; Chen et al., 2003; Hayat et al., 2010). In plants, two enzymes control the balance between SA and MeSA: the SA binding protein 2 (SABP2) that converts biologically inactive MeSA into active SA (Forouhar et al., 2005), and the SA methyltransferase 1 (SAMT1) that catalyzes the formation of MeSA from SA (Ross et al., 1999; Park et al., 2007).

Several studies provided evidence for the ability of *Botrytis* to suppress host defense by different mechanisms. These include the manipulation of plant hormone pathways, in particular those that are involved in defense responses (reviewed by Mengiste, 2012). Besides *Botrytis*, a number of plant fungi, including pathogens (e.g., *Magnaporthe oryzae, Ustilago maydis*), endophytes (e.g., *Piriformospora indica*), and mutualists (e.g., *Laccaria bicolor*) also have the ability to suppress host defense (reviewed by Rovenich et al., 2014). For example, the degradation of SA by *Aspergillus niger* was reported (Krupka et al., 1967). More recently, the biotrophic fungus *Ustilago maydis* was shown to contain a cytosolic SA hydroxylase (also called acetylsalicylate deacetylase, EC 3.1.1.55), which is able to convert SA into catechol during the infection (Rabe et al., 2013). Similarly, the fungal plant pathogen *Sclerotinia sclerotiorum* proved able to degrade SA into catechol, most presumably through the action of an endogenous SA hydroxylase (Penn and Daniel, 2013). SA hydroxylase is also predicted as being a secreted protein in the plant pathogenic fungus *Fusarium graminearum* (Brown et al., 2012). Furthermore, unconventionally secreted isochorismatase effectors of two filamentous pathogens, *Phytophthora sojae* and *Verticillium dahlia*, were shown to disrupt the plant salicylate metabolism pathway by suppressing the production of its precursor (Liu et al., 2014). Thus, an increased degradation of this molecule or an inhibition of its biosynthesis could be effective strategies for biotrophic pathogens to suppress SA-mediated defense responses.

In addition the fact that SA (Prithiviraj et al., 1997; Amborabé et al., 2002; Cory and Cory, 2005; Meyer et al., 2006; Wu et al., 2008; Qi et al., 2012; Zhou et al., 2012; Panahirad et al., 2014), acetylsalicylic acid (ASA; 2-acetoxybenzoic acid) (Alem and Douglas, 2004; Stepanović et al., 2004; Leeuw et al., 2007, 2009; Moret et al., 2007; Sebolai et al., 2008; Trofa et al., 2009; Swart et al., 2011; Zhou et al., 2012) or MeSA (Schadler and George, 2006) can directly impede fungal growth has been repeatedly reported but the mechanisms of this direct attack process are unknown.

Therefore, the function of SA and/or derivatives in the infection process appears to be complex, encompassing at least three strategies: plant defense (e.g., signalization), degradation of SA by the fungal pathogen (e.g., via a fungal SA hydroxylase or biosynthesis inhibitors) and direct fungistatic effects (e.g., growth inhibition of the pathogen). In particular, several reports pointed out an intercellular antimicrobial role for SA during *Pseudomonas* infections in *Arabidopsis* (Cameron and Zaton, 2004; Carviel et al., 2009, 2014).

It is the aim of the present work to further document the possibility that SA can repress the growth of *Botrytis*. Toward this goal, we have used a physiological approach to confirm that SA and its derivatives MeSA and acetylsalicylic acid (ASA) could inhibit *Botrytis* growth. Then a proteomics approach was used to reveal potential proteins involved in *Botrytis* growth inhibition. Proteomics is a useful complement to transcriptomics since the latter does not capture the full complexity of cellular functions (Aebersold and Mann, 2003). Indeed, a focused study on proteins can determine their level and mode of expression, post-translational modifications and the interactions they establish (Schwanhäusser et al., 2011). This approach already proved successful to characterize the proteome of mycelium tissue and the extracellular secretome from *Botrytis* (Fernández-Acero et al., 2006, 2010; Shah et al., 2009; Espino et al., 2010; Li et al., 2012; Delaunois et al., 2014; González et al., 2014; González-Fernández et al., 2014; Heard et al., 2015; for reviews on proteomics of phytopathogenic fungi, see González-Fernández and Jorrín-Novo, 2012; Bianco and Perrotta, 2015). In the present work, proteomic profiling by two-dimensional electrophoresis (2DE) in combination with mass spectrometry (MS) allowed detection and identification of statistically significant changes in the *Botrytis* proteome in the presence of different concentrations of SA or MeSA. After statistical analysis of the 2DE gels, several spots showed varying accumulation patterns in the presence of each compound, from which a number of proteins were identified by liquid chromatography coupled to tandem mass spectrometry (LC-MS/MS). As a large number of the differentially accumulated proteins in the intracellular mycelium proteome potentially corresponded to secreted proteins, we also carried out comparative analyses of the *Botrytis* extracellular secretome in the absence or presence of SA or MeSA. The present results are discussed under the possibility that the signal molecules SA and MeSA may turn antifungal and *vice versa* in plant systems.

## Materials and methods

### Biological material and culture conditions

*Botrytis* strain B05.10 was maintained on solid sporulation medium, as described by Rolland et al. (2009) and Cherrad et al. (2012). To study the mycelial radial growth, a plug of *Botrytis* mycelium was deposited at the center of a Petri dish (9 cm in diameter) containing a malt/agar medium composed of malt extract (20 g/L; Becton, Dickinson and Company), 2.0% glucose (w/v; Sigma), NH_4_Cl (0.1 M), and agar (15 g/L; Becton, Dickinson and Company) buffered at pH 5.0 or pH 7.0 (Tris-maleate 0.1 M), in the absence or presence of varying concentrations of SA, 5-sulfosalicylic acid (SSA; 2-hydroxy-5-sulfobenzoic acid), ASA (0.1 mM, 0.5 mM, 1 mM, 2.5 mM, or 5 mM) or MeSA (0.38 mM, 0.77 mM, 1.15 mM, 2.3 mM, or 5 mM), all compounds being obtained from Sigma. Mycelial radial growth was measured every day (four replicates including biological repeats). Cultures were carried out in a growth chamber thermostated at 21 ± 1°C in the dark.

For proteomic analyses, the fungus was inoculated on cellophane sheets (Biorad) by streaking 1 × 10^4^ spores gently over the surface of the membranes overlaid on the malt/agar medium described above (Shah et al., 2009; Mei et al., 2014) and transferred after 3 d on Gamborg medium (Gamborg et al., 1968) buffered at pH 5.0 (Tris-maleate 0.1M) and containing 0.1% glucose (w/v), supplemented or not with MeSA (0.38 mM) or SA (2.5 mM) during 24 h at 21°C as described by Rolland et al. (2009) and Cherrad et al. (2012). Four biological replicates were carried out per assay. To collect intracellular proteins, the mycelium on the cellophane was lyophilized during 24 h and ground twice 30 s with the disrupter/homogenizer TissueLyser II (Qiagen). Proteins were solubilized in an aqueous solution containing 4% (w/v) CHAPS (Sigma) and 1% (v/v) Protease Inhibitor Cocktail for yeast (Sigma), for 1 h at 4°C and then centrifuged at 5000 g for 10 min at 4°C. To collect the secreted proteins, the liquid medium below the cellophane sheets was recovered and submitted to a clarifying centrifugation at 4°C for 15 min at 5000 g. The corresponding supernatants were used for proteome and secretome analyses, respectively.

### Protein extractions, 2D-PAGE and densitometric gel analyses

Proteins were precipitated using trichloroacetic acid (TCA). TCA (10% w/v; Sigma) was added to the soluble proteins (intracellular mycelium proteome) or the centrifuged fungal media (extracellular secretome) and kept at 4°C overnight. Proteins were pelleted by centrifugation at 14,000 g for 15 min at 4°C and washed three times with glacial acetone (VWR Chemicals). Isoelectric focusing (IEF) was performed using the Protean IEF System (Biorad, France) according to the manufacturer's instructions. The rehydration buffer contained 8 M urea (Sigma-Aldrich), and 4% (w/v) CHAPS (Sigma). IEF was performed with 11 cm linear strips, pH 3–10 or pH 3–6 (Biorad), using the Voltage Ramp protocol recommended by the manufacturer (100 V/30 min/rapid, 250 V/30 min/linear, 1000 V/30 min/linear, 7000 V/3 h/linear, and finally 32,000 V/h (pH 3–10 IPG) or 16,000 V/h (pH 3–6 IPG) (Cherrad et al., 2012). The second dimension was carried out using the Criterion Dodeca system (Biorad). A minimum of four gels loaded with biological replicates was used for each condition. Criterion any kD TGX gels (Biorad) were run at 10°C in Laemmli buffer system (Laemmli, 1970) at 100 V for 2 h (Cherrad et al., 2012). 2D-gels were stained with silver nitrate as described (Catusse et al., 2008) then scanned and analyzed with the software SameSpots v.5 (Non-linear Dynamics Progenesis). A *t*-test of the spot volumes was calculated to compare the different treatments. Variations in spot volumes with *p* < 0.02 and fold-change >4 were considered significant.

### In-gel digestion of proteins and sample preparation for MS analysis: data acquisition and database searching

Spots were destained in 25 mM ammonium bicarbonate (NH_4_HCO_3_), 50% (v/v) acetonitrile (ACN; VWR Chemicals) and shrunk in ACN for 10 min. After ACN removal, gel pieces were dried at room temperature. Proteins were digested by incubating each gel spot with 10 ng/μL of trypsin (T6567, Sigma-Aldrich) in 40 mM NH_4_HCO_3_, 10% (v/v) ACN, rehydrated at 4°C for 10 min, and finally incubated overnight at 37°C. The resulting peptides were extracted from the gel in three steps: a first incubation in 40 mM NH_4_HCO_3_, 10% (v/v) ACN for 15 min at room temperature and two incubations in 47.5% (v/v) ACN, 5% (v/v) formic acid (Sigma) for 15 min at room temperature. The three collected extractions were pooled with the initial digestion supernatant, dried in a vacuum centrifuge (SpeedVac; Eppendorf), and resuspended with 25 μL of 0.1% (v/v) formic acid before performing the nanoLC-MS/MS analysis (Cherrad et al., 2012).

Peptide mixtures were analyzed by on-line capillary nano HPLC (LC Packings, Amsterdam, The Netherlands) coupled to a nanospray LCQ Deca XP ion trap mass spectrometer (ThermoFinnigan, San Jose, CA, USA). Ten microliters of each peptide extract were loaded on a 300 μm ID × 5 mm PepMap C18 precolumn (LC Packings, Dionex, USA) at a flow rate of 20 μL/min. After 5 min desalting, peptides were online separated on a 75 μm internal diameter × 15 cm C18 PepMapTM column (LC Packings, Amsterdam, The Netherlands) with a linear gradient of solvent B (5–40%) and solvent A (95%–60%) in 48 min (solvent A was 0.1% (v/v) formic acid in 5% (v/v) ACN, and solvent B was 0.1% (v/v) formic acid in 80% (v/v) ACN). The separation flow rate was set at 200 nL/min. The mass spectrometer operated in positive ion mode at a 1.9-kV needle voltage and a 4-V capillary voltage. Data acquisition was performed in a data-dependent mode alternating in a single run, a MS scan survey over the range *m/z* 300–1700 and three MS/MS scans with Collision Induced Dissociation (CID) as activation mode. MS/MS spectra were acquired using a 2-*m/z* unit ion isolation window, a 35% relative collision energy, and a 0.5 min dynamic exclusion duration.

Mascot and Sequest algorithms through Proteome Discoverer 1.4 Software (Thermo Fisher Scientific Inc., USA) were used for protein identification against the Broad Institute *Botrytis cinerea* database (http://www.broadinstitute.org/annotation/genome/botrytis_cinerea/MultiHome.html; 16,448 entries; Amselem et al., 2011). Two missed enzyme cleavages were allowed. Mass tolerances in MS and MS/MS were set to 2 Da and 1 Da, respectively. Oxidation of methionine and carbamidomethylation on cysteine were searched as dynamic and static modifications, respectively. Peptide validation was performed using Target Decoy PSM Validator and only “high confidence” peptides were retained corresponding to a 1% false positive rate at peptide level. The mass spectrometry proteomics data have been deposited to the ProteomeXchange Consortium (Vizcaíno et al., 2014) via the PRIDE partner repository (http://www.ebi.ac.uk/pride/help/archive/about) with the dataset identifier PXD002873.

### Bioinformatics

The Fungal Secretome Database 3.0 (Choi et al., 2010) was used to collect annotations and signal peptide prediction programs (Bendtsen et al., 2004b; Emanuelsson et al., 2007; Caccia et al., 2013). SecretomeP 2.0 (http://www.cbs.dtu.dk/services/SecretomeP/) was also used to provide information related to non-classical secretory proteins (Bendtsen et al., 2004a). Secreted proteins were classified into functional categories as described (Espino et al., 2010; Cherrad et al., 2012).

## Results

### Growth curves

The impact of salicylic acid (SA) and derivatives on mycelium growth of *Botrytis* is presented in Table 1 and Supplemental Figure S1. It appears that methylsalicylic acid (MeSA) was the most active compound in impeding fungal growth, followed by acetylsalicylic acid (ASA), and SA. In contrast 5-sulfosalicylic acid (SSA) did not entail any growth reduction (Table 1; Supplemental Figure S1). At all times of mycelial cultures and used concentrations of SA and derivatives we checked that the pH of the culture media was not affected upon MeSA, SA, ASA, or SSA addition compared to control conditions (data not shown). Based on these results, to provide clues as to the molecular mechanisms underlying the *Botrytis* response to two of the investigated compounds, MeSA and SA, we used a proteomics approach toward the characterization of the intracellular proteome and the extracellular secretome of the treated fungal cells. For these comparative proteomics experiments, we used the minimum concentration of MeSA (0.38 mM) or SA (2.5 mM) at which the smallest effect was observed on mycelial growth (Supplemental Figure S1). This protocol allows observation of the early events of the inhibition process and to minimize cell death and possible cell lysis that would complicate the analysis of the extracellular secretome.

**Table 1:** **Growth rates observed at 6 day of mycelium culture in the presence of MeSA, SA, ASA, or SSA**.

**Compound (5 mM)**	**Relative growth rate (%) (6 d)**
MeSA	6.2 ± 2.6
SA	53.7 ± 15.2
ASA	23.4 ± 12.2
SSA	100.8 ± 3.5

*Measurements of mycelium growth (four replicates) in control conditions or in the presence of 5 mM MeSA, SA, ASA, or SSA were taken at 6 d of the cultures as detailed in Materials and Methods. Standard deviations are shown*.

### Intracellular proteome

#### Effect of MeSA

**Figure 1:**
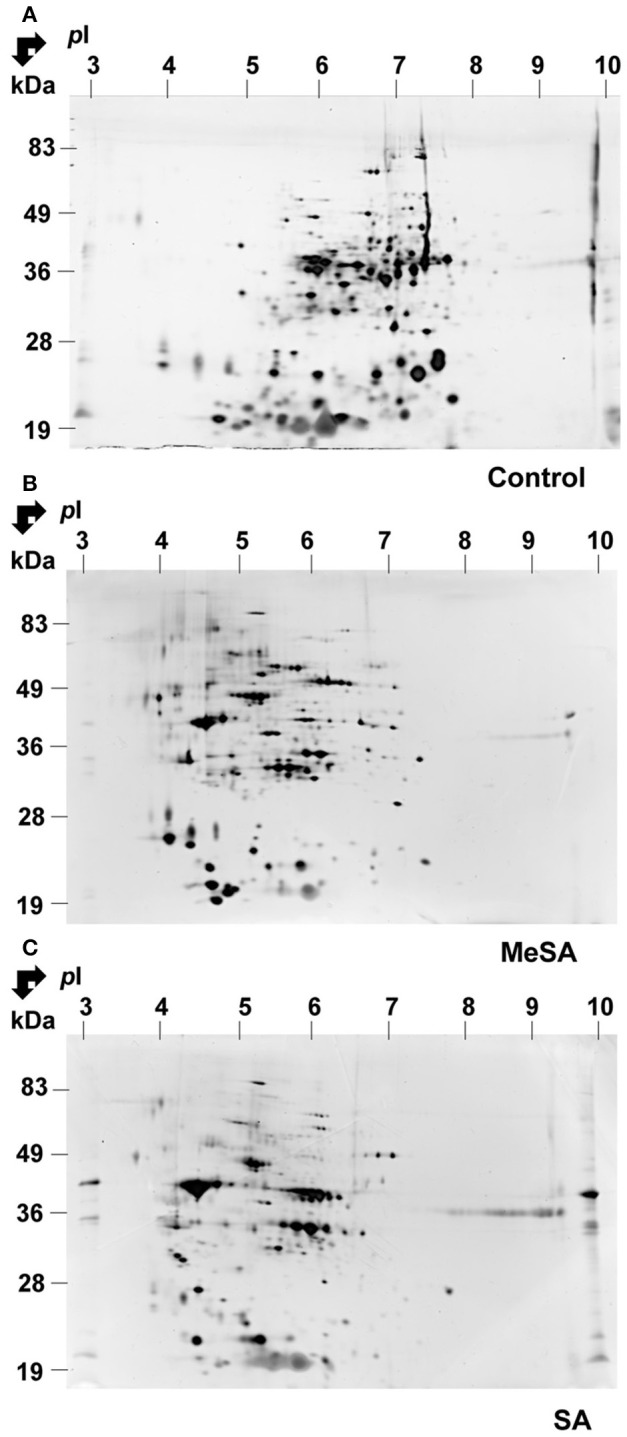
**Effect of MeSA or SA on the intracellular *Botrytis* proteome**. Protein extracts corresponding to the intracellular mycelium proteome were prepared as described in Materials and Methods, and subjected to 2DE in untreated control conditions **(A)** or in the presence of MeSA at 0.38 mM **(B)** or SA at 2.5 mM **(C)** (four replicates in each condition). Following densitometric analyses of the 2D gels as described in Materials and Methods, differentially accumulated spots (*p* < 0.02; 4-fold change) were submitted to MS analysis and the proteins characterized were listed in Supplemental Table S1.

A typical 2D gel obtained for the MeSA-treated mycelium is shown in Figure 1B. By visual inspection, there was a major impact of MeSA on the mycelium proteome. Thus, a number of basic spots disappeared from the control accompanied by an increase in the number of acidic spots in the MeSA-treated proteome (compare Figures 1A,B). This was confirmed by global densitometric analyses of the 2D gels (Supplemental Figure S2).

**Figure 2:**
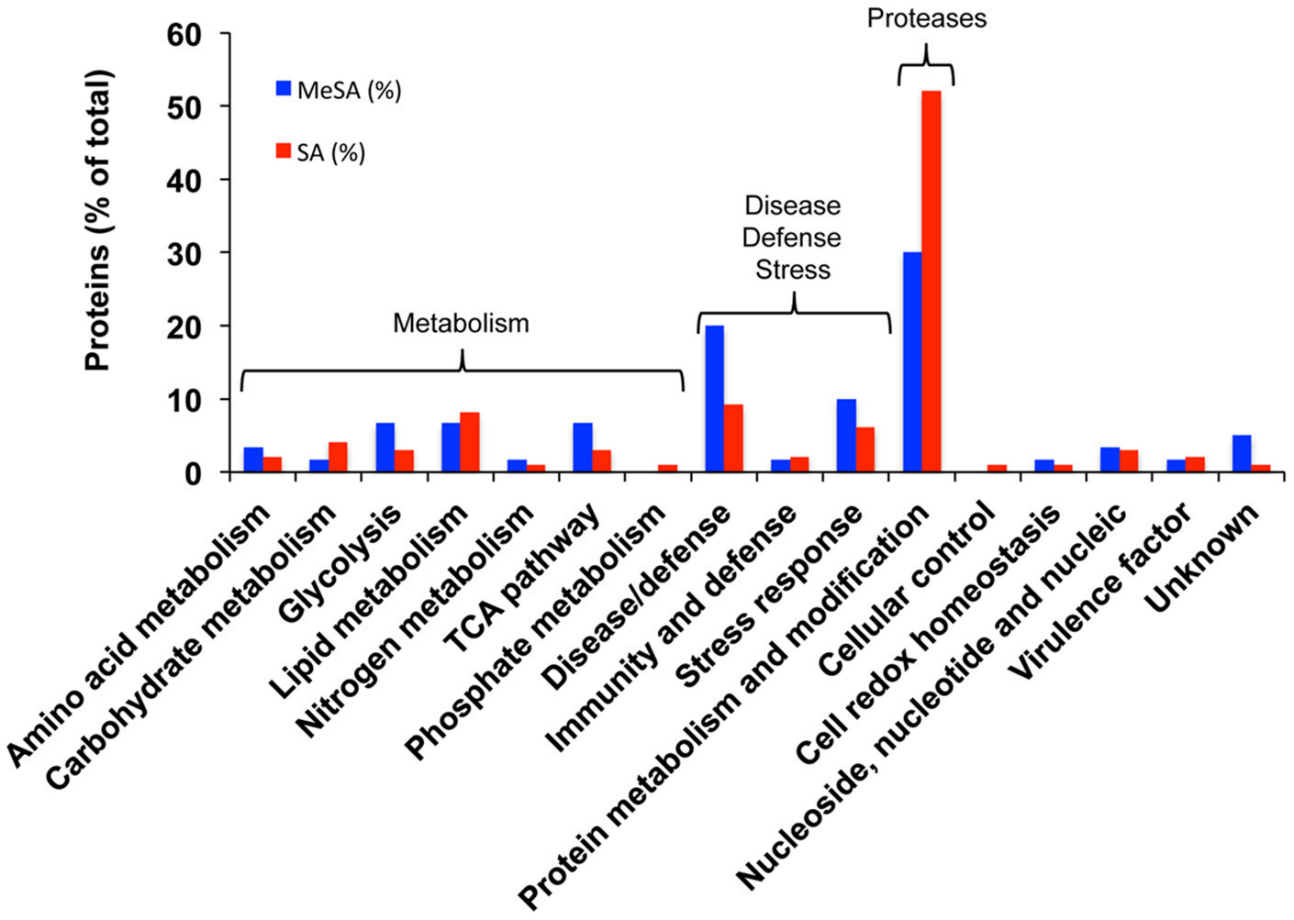
**Functional categorization of the proteins found in differentially accumulated spots of the *Botrytis* mycelial proteomes obtained in the presence of MeSA (0.38 mM, blue) or SA (2.5 mM, red) compared to control untreated mycelium**. Blue bars, MeSA-treated mycelium; red bars, SA-treated mycelium. The results are expressed in % values of total differentially accumulated proteins, (*p* < 0.02; 4-fold change), namely 60 proteins for MeSA and 98 proteins for SA (Supplemental Table S1).

Densitometric analyses of the 2D-gels from MeSA-treated vs. control mycelium (Figures 1A,B; four replicates) revealed that the volumes of 48 spots varied (*p* < 0.02; 4-fold change) (Supplemental Table S1; Supplemental Figure S3), of which 37 contained a single protein, 10 contained two proteins, and one contained three proteins for a total of 60 proteins (Supplemental Table S1). The largest functional category comprised 19 proteins (31.7%) and was associated with disease/defense, immunity/defense, and stress response mechanisms (collectively referred to as disease/defense/stress in Figure 2). The second and third largest functional categories were each composed of 18 proteins (30%). They were associated with the protein metabolism and modification category and with enzymes involved in various metabolic processes collectively referred to as metabolism in Figure 2 of which 14 corresponded to various proteases (Supplemental Table S1; Figure 2; Table 2).

**Table 2:** **Differentially accumulated intracellular proteins in the presence of MeSA or SA in the *Botrytis* culture medium compared to untreated control fungal cells**.

**Accession**	**Description/Functional classification**
**Amino acid metabolism**	
BC1G_00474	Fumarylacetoacetate hydrolase
**Glycolysis**	
BC1G_08882	Triosephosphate isomerase
BC1G_11392	2,3-Bisphosphoglycerate-independent phosphoglycerate mutase
BC1G_12178	Glucokinase GLK1
**TCA pathway**	
BC1G_11376	Dihydrolipoyl dehydrogenase, mitochondrial precursor
BC1G_16294	Aconitase/homoaconitase
**Lipid metabolism**	
BC1G_02986	Phosphatidylglycerol/phosphatidylinositol transfer protein
BC1G_06765	Enoyl-[acyl-carrier-protein] reductase 1
**Nitrogen metabolism**	
BC1G_08982	Nitrilase family protein (Nit3)
**Phosphate metabolism**	
BC1G_02965	Acid phosphatase
**Protein metabolism and modification**	
BC1G_01026	Tripeptidyl-peptidase 1
BC1G_02223	Protein disulfide isomerase
BC1G_06849	Vacuolar protease A
BC1G_07068	Aspergillopepsin A
BC1G_09731	Elongation factor 2
BC1G_02944	Tripeptidyl-peptidase 1
BC1G_03070	Rhizopuspepsin-2
BC1G_03711	Serine carboxypeptidase 3
BC1G_06836	Subtilase-type proteinase psp3
**Stress response**	
BC1G_04390	DnaK-type molecular chaperone BiP
BC1G_06164	Heat shock protein 70 kDa
BC1G_08723	IN2-2 protein, Aldo-keto reductase
BC1G_09341	Heat shock protein 60
**Disease/defense**	
BC1G_08946	Cyanate hydratase
BC1G_01910	Manganese superoxide dismutase
BC1G_06362	Aldehyde dehydrogenase
BC1G_08301	Ascorbate peroxidase
BC1G_12146	Catalase
**Immunity and defense**	
BC1G_12374	IgE-binding protein
**Cellular control**	
BC1G_10630	Sporulation-specific protein 2
**Unknown**	
BC1G_07825	Predicted protein

*Only the proteins present in differentially accumulated spots containing a single protein are listed. For other details, see Supplemental Table S1*.

**Figure 3:**
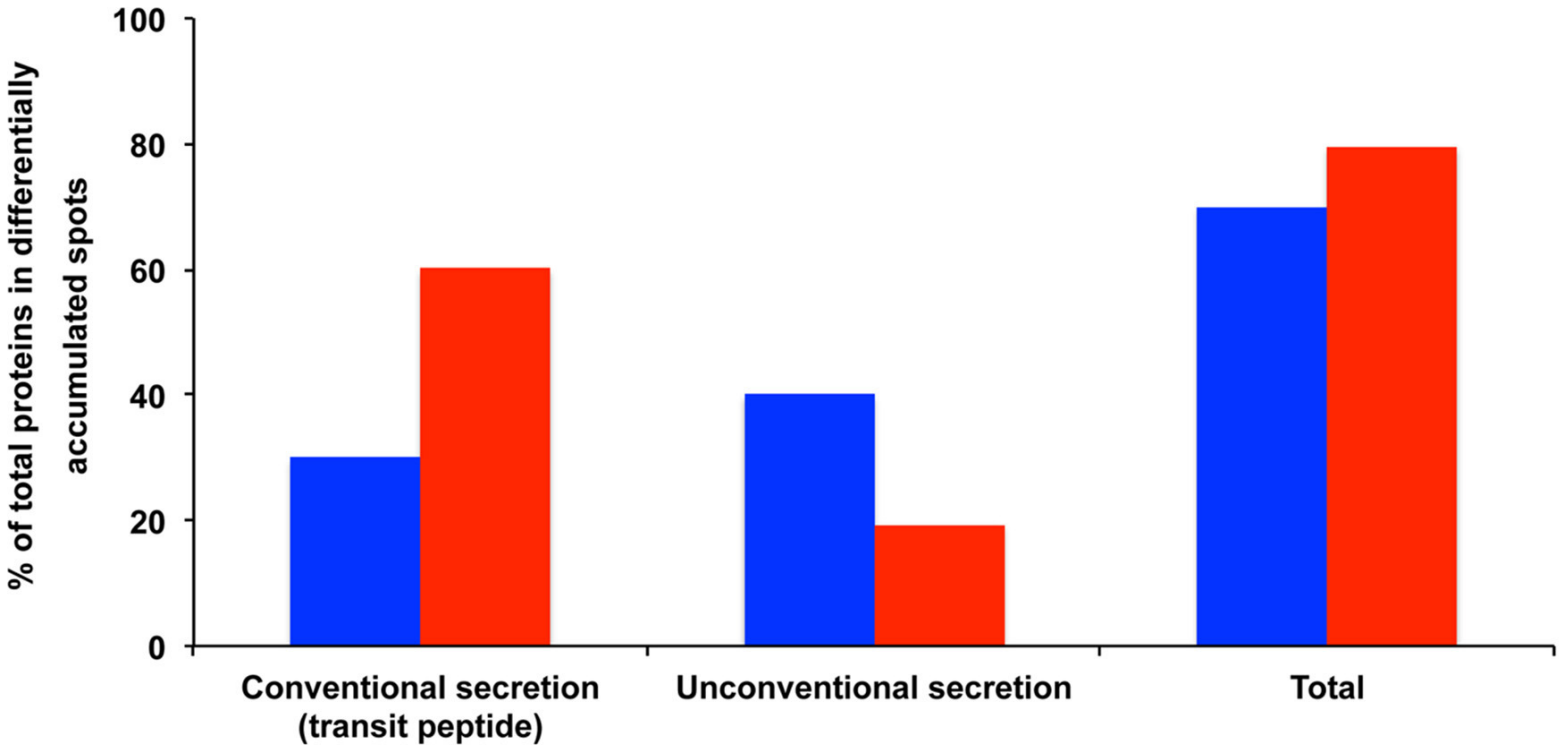
**Potentially secreted proteins found in protein spots exhibiting varying volumes in MeSA- or SA-treated intracellular mycelial proteomes**. The data show the proportion of proteins predicted to be secreted by conventional secretion (i.e., those proteins that contain a transit peptide as predicted by TargetP and SignalP) or through unconventional secretion (i.e., those proteins that do not contain a transit peptide but are predicted to be secreted by SecretomeP) (Supplemental Table S1). The results are expressed as percentages of total differentially accumulated proteins, namely 60 proteins (blue bars) and 98 proteins (red bars) for the MeSA- and SA-treated intracellular mycelial proteomes, respectively (see Supplemental Table S1).

Out of the 60 proteins found in the 48 differentially accumulated spots, 18 (30%) exhibited a transit peptide. Yet SecretomeP predicted that 24 transit-peptide-devoid proteins could be secreted through non-canonical secretion pathways (Nickel, 2003). Thereby in total a large proportion (70%) of the proteins found in the differentially accumulated spots in the presence of MeSA corresponded to putatively secreted proteins (Supplemental Table S1; Figure 3).

The same trends were observed for the 37 proteins obtained from differentially accumulated spots containing a single protein (Supplemental Table S1). The largest functional categories corresponded to protein metabolism and modification (16 proteins; 43.2%); proteins involved in various metabolic processes (11 proteins; 29.7%); and disease/defense, immunity/defense, and stress response mechanisms (nine proteins; 24.3%; Table 2; Supplemental Table S1). Sixteen proteins present in differentially accumulated spots containing a single protein were predicted to contain a transit peptide (TargetP, SignalP). Furthermore, SecretomeP predicted that 13 transit-peptide-devoid proteins could be secreted through non-canonical secretion pathways. Again, a large proportion (78.4%) of the MeSA-responsive proteins present in unique spots corresponded to putatively secreted proteins (Supplemental Table S1).

#### Effect of SA

As for MeSA, the volume of a large the number of acidic spots increased in the SA-treated intracellular proteome, which was accompanied by a decreased number of basic spots from the control (compare Figures 1A,C; Supplemental Figure S2). Densitometric analyses of the 2D gels (Figures 1A,C; four replicates) revealed that the volumes of 60 spots varied (*p* < 0.02; 4-fold change; Table 2; Supplemental Figure S3; Supplemental Table S1), of which 33 contained a single protein, 19 contained two proteins, six contained three proteins, one contained four proteins, and one contained five proteins, for a total of 98 proteins.

As for MeSA, the three largest functional categories corresponded to protein metabolism and modification (51 proteins; 52%) of which 45 corresponded to various proteases; various proteins involved in metabolism (25 proteins; 25.5%); and disease/defense, immunity/defense, and stress response mechanisms (17 proteins; 17.3%) (Supplemental Table S1; Figure 2).

Out of the proteins found in the differentially accumulated spots, 59 (60.2%) exhibited a transit peptide. Furthermore, SecretomeP predicted that 19 proteins not predicted to contain a transit peptide could be secreted through non-canonical secretion pathways. Thereby, as in the case of MeSA a large proportion (80.6%) of the proteins found in differentially accumulated spots in the presence of SA corresponded to putatively secreted proteins (Supplemental Figure S1; Figure 3).

The same analysis was conducted for the 33 proteins present in spots containing a single protein. The three largest functional categories corresponded to protein metabolism and modification (12 proteins; 36.4%); metabolism (11 proteins; 33.3%); and disease/defense, and stress response mechanisms (nine proteins; 27.3%) (Table 2; Supplemental Table S1). Out of these 33 proteins, 14 were predicted to contain a transit peptide. Moreover, SecretomeP predicted that 10 proteins not predicted to contain a transit peptide could be secreted through non-canonical secretion pathways (Supplemental Table S1). Again, a large proportion (72.7%) of the SA-responsive proteins present in unique spots corresponded to putatively secreted proteins (Table 2; Supplemental Table S1).

### Extracellular secretome

**Figure 4:**
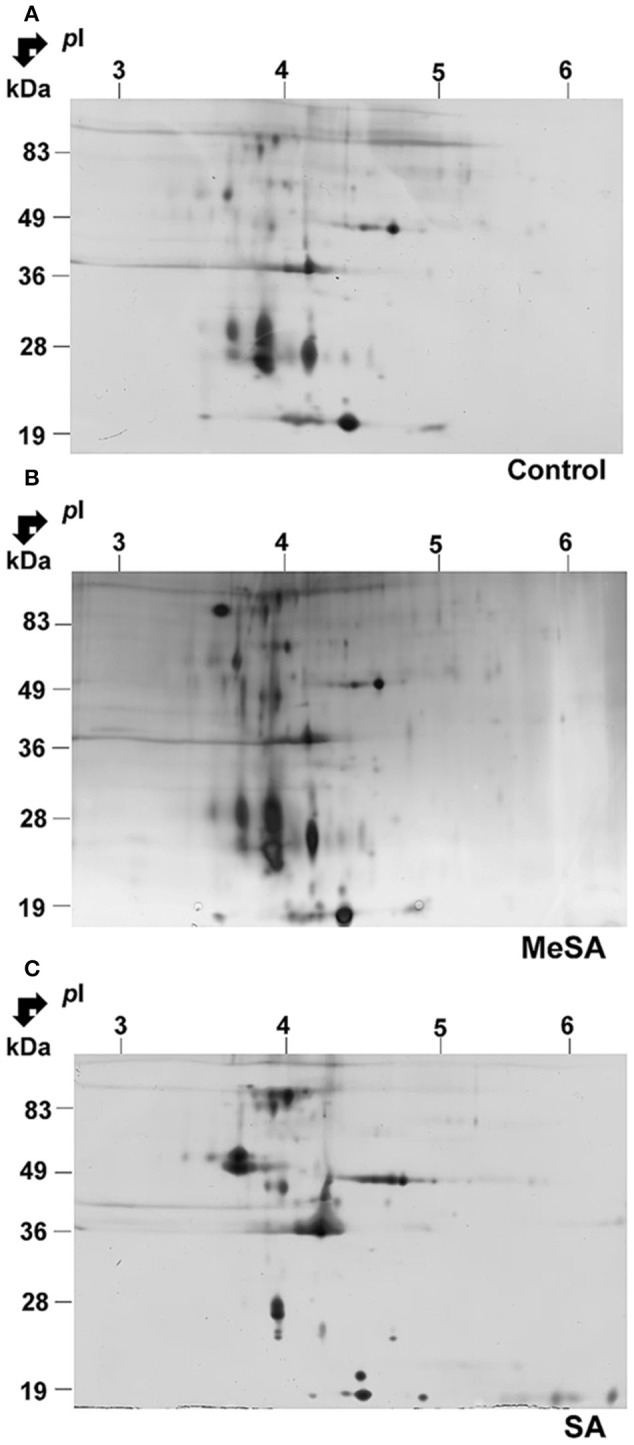
**Effect of MeSA or SA on the extracellular *Botrytis* secretome**. Protein extracts corresponding to the extracellular mycelium secretome were prepared as described in Materials and Methods, and subjected to 2DE in control untreated conditions **(A)** or in the presence of MeSA at 0.38 mM **(B)** or SA at 2.5 mM **(C)** (four replicates in each condition). Following densitometric analyses of the 2D gels as described in Materials and Methods, differentially accumulated spots (*p* < 0.02; 4-fold change) were submitted to MS analysis and the proteins characterized were listed in Supplemental Table S2.

The above findings suggested that a large proportion of proteins found in differentially accumulated spots of the intracellular proteomes corresponded to potentially secreted proteins (Supplemental Table S1; Figure 3). To assess whether such modifications in intracellular protein abundance could be reflected at the level of the corresponding fungal extracellular secretomes, we prepared protein extracts from the extracellular growth media as described in Materials and Methods. Here the extracellular secretome corresponds to the proteins found in the liquid medium below the cellophane membrane on which fungal cells are grown. This prevents the contamination of the extracellular secretome (as presently defined) by intact fungal cells. Furthermore, growing fungal cells on a solid surface rather than a liquid media better reflects the conditions under which *Botrytis* infections of plants naturally occur (Shah et al., 2009). Typical gels of the extracellular secretomes obtained in such conditions are shown in Figure 4. These gels appeared to have a somewhat lower resolution than those obtained for the intracellular proteomes (Figure 4). In fact, when the extracellular secretomes of plant fungal pathogens or free-living fungi were analyzed in previous studies such behavior was repeatedly observed (Medina et al., 2005; Oda et al., 2006; Cobos et al., 2010; Espino et al., 2010; Fernández-Acero et al., 2010; Lu et al., 2010; Jung et al., 2012; Yang et al., 2012; González et al., 2013, 2014; Fernandes et al., 2014; Gómez-Mendoza et al., 2014). One reason could be the high amount of polysaccharides and the presence of low-molecular-weight metabolites in fungal secretomes (Chevallet et al., 2007; Erjavec et al., 2012; Fernandes et al., 2014). These molecules are known to interfere with protein extraction and separation methods (Lemos et al., 2010). Despite this difficulty, differentially accumulated spots could be revealed by image analysis of 2D gels upon MeSA or SA treatments of the *Botrytis* mycelia (Figure 4; Supplemental Table S2).

In the presence of MeSA six spots were differentially accumulated (Supplemental Table S2; Supplemental Figure S4). Two contained a single protein, one contained two proteins and two contained three proteins, for a total of 10 proteins. They all possessed a transit peptide (Supplemental Table S2). The observed functional categories were cellular control, carbohydrate metabolism, amino acid metabolism, and immunity and defense (Supplemental Table S2).

In the presence of SA, 22 spots exhibited significant volume variations with respect to control (Supplemental Figure S4). Among them, 16 contained a single protein (Table 3), four contained two proteins, and two contained three proteins, for a total of 30 proteins (Supplemental Table S2), of which 25 (83.3%) possessed a transit peptide (Supplemental Table S2). Furthermore, SecretomeP predicted that two of proteins that did not exhibit a transit peptide could be secreted through non-canonical secretion pathways (Supplemental Table S2). Thus, in total as much of 90% of the proteins found in differentially accumulated spots in the presence of SA were predicted as being secreted. These were distributed in functional categories corresponding to carbohydrate metabolism, cell redox homeostasis, cellular control, disease/defense, protein metabolism, and modifications (Supplemental Table S2).

**Table 3:** **Differentially accumulated extracellular proteins in the presence of MeSA or SA in the *Botrytis* culture medium compared to untreated control fungal cells**.

**Accession**	**Description/Functional classification**
**Protein metabolism and modification**	
BC1G_03070	Rhizopuspepsin-2
BC1G_07068	Aspergillopepsin A
BC1G_09180	Penicillolysin
**Immunity and defense**	
BC1G_12374	IgE-binding protein
**Cellular control**	
BC1G_10630	Sporulation-specific protein 2
**Cell redox homeostasis**	
BC1G_14403	Thioredoxin
**Carbohydrate metabolism**	
BC1G_14030	Beta glucanosyltransferase
**Virulence factor**	
BC1G_02163	Cerato-platanin protein
**Unknown**	
BC1G_00896	Predicted protein
BC1G_07825	Predicted protein

*Only the proteins present in differentially accumulated spots containing a single protein are listed. For other details, see Supplemental Table S2*.

## Discussion

In good agreement with previous studies showing that SA (Prithiviraj et al., 1997; Amborabé et al., 2002; Cory and Cory, 2005; Meyer et al., 2006; Wu et al., 2008; Qi et al., 2012; Zhou et al., 2012; Panahirad et al., 2014), ASA (Alem and Douglas, 2004; Stepanović et al., 2004; Leeuw et al., 2007, 2009; Moret et al., 2007; Sebolai et al., 2008; Trofa et al., 2009; Swart et al., 2011; Zhou et al., 2012) or MeSA (Schadler and George, 2006) can directly impede growth in several fungal species, the present study documents that of the four compounds analyzed (ASA, MeSA, SA, SSA) three of them (SA, ASA, and MeSA) showed fungistatic activity toward *Botrytis*. That SSA was not active in blocking *Botrytis* growth also agrees with the absence of reports reporting a fungistatic activity for this molecule. Very interestingly, several studies also reported that in addition to its role as a signaling molecule SA can also alter *in vivo* the growth of various microorganisms in interaction with plants. Thus, previous work in *Arabidopsis* indicated that accumulation of SA in the intercellular space is an important component of basal/PAMP-triggered immunity as well as effector-triggered immunity to pathogens that colonize the intercellular space (Cameron and Zaton, 2004; Carviel et al., 2009, 2014). The present data are also in good agreement with previous studies on the effect of SA on symbiotic root microbiomes both in bacteria and fungi (Medina et al., 2003; Stacey et al., 2006) and with recent results showing that plant SA, as well as exogenously applied SA, can help sculpt root microbiome by modulating colonization of the root by specific bacterial families (Lebeis et al., 2015).

Phenolic compounds, sodium salicylate, and related compounds have been reported to inhibit tumor cell growth in mouse leukemia L1210 cells (Cory and Cory, 2005). Interestingly, this study revealed that the IC_50_ values (half maximal inhibitory concentrations) determined for these compounds correlated extremely well with the apparent ability of the drugs to enter the cells as estimated by the ratio of octanol-aqueous distribution (Leo et al., 1971; Unger et al., 1978); in particular this octanol-aqueous distribution accounted for the very low activity observed with SSA compared to that measured for SA (Cory and Cory, 2005). In addition, it is known that SA methylation increases its membrane permeability, as well as its volatility, thus allowing more effective long distance transport of this defense signal (Dempsey et al., 2011). Hence a likely explanation for the observed differences in the ability of these compounds to inhibit *Botrytis* growth could be related to their relative efficiencies to penetrate into the fungal cells.

Knowing the existence of enzymes catalyzing the conversion of ASA or MeSA into SA, one may wonder whether these SA derivatives could directly inhibit *Botrytis* growth or if their action resulted from their conversion to SA or to MeSA. ASA esterase (EC 3.1.1.55), which catalyzes the hydrolysis of ASA to yield SA and acetate, has been widely described in animals and human (Spenney and Nowell, 1979; Ali and Kaur, 1983; White and Hope, 1984; Kim et al., 1990), but there are no reports on the existence of this enzyme in plants or fungi. In agreement BLAST searches against the *Botrytis* genome did not confirm the existence of such an enzyme in this fungus (data not shown). In the case of MeSA, the tobacco methylsalicylate esterase SABP2 (EC 3.1.1.-), a 29-kDa protein, catalyzes the conversion of MeSA into SA to induce SAR (Kumar and Klessig, 2003; Forouhar et al., 2005; Tripathi et al., 2010). While BLAST searches confirmed the existence of SABP2 in various plant species they did not support the existence of such an enzyme in fungal species, notably in *Botrytis* (data not shown). Also, BLAST searches did not support the existence in *Botrytis* of a SA methyltransferase (SAMT 1) analogous to that found in tobacco (Ross et al., 1999; Park et al., 2007) (data not shown). The results therefore suggest that ASA, MeSA and SA are active *per se* in *Botrytis* growth inhibition.

We further show that MeSA and SA treatments substantially modify the *Botrytis* intracellular and extracellular mycelial proteome. In the following we discuss some specific aspects of the observed modifications.

### *p*I SHIFT

For both SA- and MeSA-treated intracellular mycelium proteomes we observed a large *p*I shift in the localization of the revealed spots on 2D gels. Thus, the addition of either of these two molecules in the *Botrytis* culture medium was accompanied with an accumulation of spots located in the acidic *p*I range of the 2D gels, while there was a decreased number of protein spots located in a more basic region of the 2D gels (Figure 2). As a somewhat similar behavior was noted when changing the pH of the culture medium from 5.0 to 7.0 (Supplemental Figure S2), it is possible that at least part of the effects of SA and MeSA reflects a change in pH regulation in *Botrytis*. Many fungi grow over a wide pH range and their gene expression is tailored to the environmental pH. In *Aspergillus nidulans*, the transcription factor PacC, an activator of genes expressed in alkaline conditions and a repressor of those expressed in acidic conditions, undergoes two consecutive proteolytic events, the first being pH-signal dependent and the second proteasomal (Peñalva et al., 2008). In previous work we suggested a possible link between pH regulation and metal response in *Botrytis* (Cherrad et al., 2012). Consistent with this, it has been documented that Rim101 (the ortholog of PacC in yeasts) and PacC are involved in metal (iron or zinc) homeostasis in yeasts (Conde e Silva et al., 2009; Ariño, 2010; Linde et al., 2010) and filamentous fungi such as *Aspergillus fumigatus* (Amich et al., 2009; Cherrad et al., 2012). Furthermore, in *Aspergillus nidulans*, biosynthesis and uptake of siderophores are regulated not only by iron availability but also by ambient pH through the transcription factor PacC (Eisendle et al., 2004). In this context, it is therefore of interest to note that SA and its derivatives can form chelate compounds with metal ions (Perrin, 1958). Hence an alteration in metal homeostasis could provide an explanation for the observed changes in the intracellular proteomes in the presence of MeSA or SA.

Although in our experiments the extracellular pH was not affected upon addition of ASA, MeSA, or SSA to the culture medium, we cannot rule out the possibility that accumulation of these molecules within *Botrytis* cells entailed a modification of the intracellular pH that would have been perceived by the PacC regulatory system. Further, work is needed to address this question.

### Metabolism

Nulton-Persson et al. (2004) reported that treatment of isolated cardiac mitochondria with SA or ASA resulted in alterations of mitochondrial respiration, most presumably through inhibition of the Krebs cycle complex alpha-ketoglutarate dehydrogenase (KGDH; EC 1.2.4.2). It is therefore interesting to note that mitochondrial dihydrolipoyl dehydrogenase (EC 1.8.1.4), the E3 component of the KGDH complex, was strongly up accumulated in the SA- and ASA-treated intracellular mycelial proteomes (Supplemental Table S1). It is noted also that the accumulation of aconitase (one another enzyme in the Krebs cycle; EC 4.2.1.3) was modified in the SA- and ASA-treated intracellular mycelial proteomes (Supplemental Table S1). Since a number of fungicides behave as very potent inhibitors of mitochondrial respiration (Gisi et al., 2002; Avenot and Michailides, 2010; Belenky et al., 2013; Sierotzki and Scalliet, 2013), one possibility to account for the inhibition of *Botrytis* growth in the presence of MeSA, ASA or SA could rely on perturbation of mitochondrial respiration.

### Proteases

For the MeSA-treated mycelium 14 proteins corresponded to various proteases (Supplemental Table S1; Figure 2) corresponding to five different enzymes, BC1G_02949 (tripeptidyl-peptidase 1), BC1G_03070 (rhizopuspepsin-2), BC1G_03711 (serine carboxypeptidase 3), BC1G_06836 (subtilase-type proteinase psp3), and BC1G_06849 (vacuolar protease A). For the SA-treated intracellular mycelial proteome, the main functional category of the proteins found to be present in the highly accumulating spots corresponded to proteases (Supplemental Table S1). Most of them (88.8%) were predicted as being secreted and corresponded to six types of proteases, namely BC1G_01026 and BC1G_02944 (tripeptidyl-peptidase 1, a serine protease), BC1G_03070 (rhizopuspepsin-2, an aspartic protease), BC1G_03711 (serine carboxypeptidase 3, a serine protease), BC1G_06320 (dipeptidase 1, a zinc metallopeptidase), BC1G_06836 (subtilase-type proteinase psp3, a serine protease), and BC1G_06849 (vacuolar protease A, an aspartic protease) (Supplemental Table S1; Figure 2; http://merops.sanger.ac.uk/cgi-bin/speccards?sp=sp001886;type=peptidase). Five of the presently characterized proteases (BC1G_01026, BC1G_02944, BC1G_03070, BC1G_06836, and BC1G_06849) have previously been found in the extracellular *Botrytis* secretome (Espino et al., 2010; Fernández-Acero et al., 2010; Li et al., 2012; González-Fernández et al., 2014). Of these, only BC1G_03070 was presently found in differentially accumulated spots of the extracellular secretome in the presence of SA (Supplemental Table S2). One possibility to explain this behavior could rely on the different growing conditions used in the present work and in previous studies aiming at characterizing the *Botrytis* secretome. As stressed by Espino et al. (2010) the *Botrytis* secretome is highly adaptive, in the sense that very different sets of proteins are detected in the secretome when the growth conditions, or the age of the mycelium, differ. In particular these authors observed that only three proteins from a total number of 238 experimentally characterized extracellular proteins are present in 10 different experimental conditions, implying that *Botrytis* can greatly alter the composition of the secreted protein pool to meet the requirement of the different growing needs (Espino et al., 2010).

Besides the well-established role of extracellular proteolytic activity in fungal pathogenicity (Naglik et al., 2003; ten Have et al., 2010; Jashni et al., 2015), intracellular proteinases were also shown to play important functional roles in fungi (Li and Kane, 2009). In particular, vacuolar proteases have been reported to be essential in morphogenesis and adaptation to ambient nutritional conditions (Yike, 2011). We note that BC1G_06849 presently found in the intracellular proteome of *Botrytis* cells treated with either SA or MeSA (Supplemental Table S1) is predicted to encode a vacuolar protease (ten Have et al., 2010).

### ROS detoxification

Three enzymes involved in ROS detoxification, namely catalase (BC1G_12146; EC 1.11.1.6), ascorbate peroxidase (BC1G_08301; EC 1.11.1.11), manganese superoxide dismutase (BC1G_01910; EC 1.15.1.1) and peroxiredoxin (BC1G_09932; EC 1.11.1.15) were strongly depressed in the intracellular mycelium proteome upon MeSA or SA addition (Supplemental Table S1). Therefore, it is possible that the reduction of fungal growth observed in the presence of these compounds arose from an increased ROS accumulation resulting in an oxidative stress. It is worth noting that in plants SA specifically inhibits the H_2_O_2_-degrading activity of catalase (Chen et al., 1993) and of ascorbate peroxidase (Durner and Klessig, 1995), while SA treatment induces an increase in H_2_0_2_ concentrations *in vivo*, suggesting that SA may facilitate H_2_O_2_ accumulation during the oxidative burst induced by infection with avirulent pathogens (reviewed by Vlot et al., 2009). It is therefore very interesting to note that SA could target the same enzymes in plants and phytopathogenic fungi.

### Cell wall remodeling and integrity

An analysis of the intracellular mycelium proteome revealed that spots containing the cerato-platanin related protein (CPP) had reduced volumes upon SA addition to the culture medium (Supplemental Figure S1). The CPPs, originally discovered in *Ceratocystis fimbriata* f. sp. *platani* (Pazzagli et al., 1999), are elicitors or effector proteins that belong to a larger class of fungal proteins defined as SSCPs or SSPs: small, secreted (cysteine rich) proteins (Lamdan et al., 2015). Thus, Frías et al. (2011, 2013, 2014) showed that BcSpl1, a member of the CPP family, is required for full virulence in *Botrytis* and elicits the hyper sensitive response in the plant host. Also, ectopic expression of the *Magnaporthe oryzae* CPP gene, *MgSM1*, upregulates the expression of plant defense genes such as PR-1, PR-5, and PDF1.2 and induces local hypersensitivity reactions (Yang et al., 2009). It is therefore very interesting to note that the same SA molecule can both elicit HR in plants and alter the accumulation of CPPs in phytopathogenic fungi. Presumably this would allow to fine tune the regulation of HR during plant infection by phytopathogenic fungi. CPPs are structurally related to expansins, which are proteins associated with carbohydrate binding and loosening of the cellulose scaffolds in plant cell walls (de Oliveira et al., 2011). Therefore in addition to such a role in virulence, fungal CPPs might also be involved in growth and development, which should justify their presence in the fungal cell wall where they could act by disrupting non-covalent interactions between fungal cell wall components: for example, between β-glucan or chitin chains (Gaderer et al., 2014; Baccelli, 2015). In this way CPPs could act in all those processes requiring remodeling and enlargement of the fungal cell wall (Baccelli, 2015). Our present observation might support such a role, thus accounting for *Botrytis* growth reduction in the presence of MeSA and SA. It is also interesting to note that the accumulation of the *Botrytis* Bcspl1 CPP (BC1G_02163) dramatically increased in the extracellular secretome in the presence of plant extracts (Shah et al., 2009), suggesting that the accumulation of this protein in the extracellular secretome is modulated by the interaction between plant and fungal cells.

Another protein revealed in this work concerned the sporulation-specific protein 2 (BC1G_10630) that corresponds to the GPI-anchored cell wall organization protein ECM33 protein of *Erysiphe necator* (Pardo et al., 2004). This protein was detected in spots showing increased volumes in the intracellular and extracellular proteomes of SA-treated *Botrytis* cells (Supplemental Table S1). This cell wall protein ECM33 was shown to be important for cell wall integrity in term of correct assembly of the mannoprotein layer (Pardo et al., 2004; Chabane et al., 2006; Martinez-Lopez et al., 2006). The present results suggest that the observed alterations in the accumulation of this protein in response to SA or MeSA treatment may represent a compensatory response to reduced cell wall integrity in *Botrytis*.

## Conclusion

In the present work, we studied the impact of SA and SA derivatives on *Botrytis* growth. By using proteomics we revealed several potential mechanisms that could account for the observed fungal growth inhibition, notably pH regulation, metal homeostasis, mitochondrial respiration, ROS accumulation, and cell wall remodeling. During infection there is ample circumstantial evidence that the plant host synthesizes SA as a signaling molecule to induce the HR response. Apoplastic SA concentrations in tobacco can be quite high at the HR lesions (Huang et al., 2006). Provided that the actual SA (or MeSA) concentrations at the infection points are high enough the present observations support a role played by the phytohormone SA and derivatives in directly containing the pathogens. As stressed by Vlot et al. (2009) SA is indeed a multifaceted hormone to combat disease.

## Conflict of interest statement

The authors declare that the research was conducted in the absence of any commercial or financial relationships that could be construed as a potential conflict of interest.

## Supplementary material

Supplemental Figure S1 **Relative growth of *Botrytis* in the absence (control) or presence of the indicated compounds (ASA, SA, MeSA, and SSA)**. Measurements of mycelium growth (four replicates) were effected at 3 d of the cultures as detailed in Materials and Methods.

Supplemental Figure S2 **Distribution of the total absorbance in acidic (1) and basic (2) regions of 2D gels for intracellular mycelium proteome. (A)** Acidic and basic regions of 2D gels. Gels shown corresponded to untreated mycelium grown at pH 5.0 (Control pH 5.0) or at pH 7.0 (Control pH 7.0) or to MeSA-treated mycelium grown at pH 5.0 (MeSA pH 5.0) or to SA-treated mycelium grown at pH 5.0 (SA pH 5.0). The acidic region (1) corresponded to the 3.0–6.0 *p*I range and the basic region corresponded to the 6.0–10.0 *p*I range of the 2D gels (separated by the blue lines). **(B)** Measurements of total absorbance in regions 1 and 2 of the 2D gels shown in **(A)**. The total absorbance in each of the two regions was measured using the software Mesurim (http://acces.ens-lyon.fr/acces/logiciels/mesurim/guide-dutilisation/mesures-sur-limage#lumsurf) and three replicates for each condition. The ratios of total absorbance in region 2/total absorbance in region 1 are listed.

Supplemental Figure S3 **Spot numbering for spots showing variations in spot volumes in the *Botrytis* intracellular mycelium proteome treated with MeSA or SA**. The differentially accumulated spots are depicted on a typical 2D-gel corresponding to the intracellular proteome of untreated *Botrytis* control: Red arrows, differentially accumulated spots from MeSA-treated mycelium; green arrows, differentially accumulated spots from SA-treated mycelium; blue arrows, differentially accumulated spots from both MeSA- and SA-treated mycelium. The proteins contained in the various differentially accumulated spots are listed in Supplemental Table S1.

Supplemental Figure S4 **Spot numbering for spots showing variations in spot volumes in the *Botrytis* extracellular mycelium secretome upon mycelium treatment with MeSA (0.38 mM) or SA (2.5 mM)**. The differentially accumulated spots are displayed on a typical 2D-gel corresponding to the extracellular secretome of control untreated *Botrytis*: Red arrows, differentially accumulated spots from MeSA-treated mycelium; green arrows, differentially accumulated spots from SA-treated mycelium; blue arrows, differentially accumulated spots from both MeSA- and SA-treated mycelium. The proteins contained in the various differentially accumulated spots are listed in Supplemental Table S2.

Supplemental Table S1 **List of intracellular proteins present in differentially accumulated spots upon comparing control untreated and MeSA- or SA-treated *Botrytis* mycelium**. MeSA or SA concentrations were at 0.38 mM or 2.5 mM, respectively.

Supplemental Table S2 **List of proteins present in differentially accumulated spots of extracellular secretomes upon comparing control untreated and MeSA- or SA-treated *Botrytis* mycelium**. MeSA or SA concentrations were at 0.38 mM or 2.5 mM, respectively.

## Abbreviations

2D: two dimensional
ACN: acetonitrile
ASA: acetylsalicylic acid
CHAPS: 3-[(3-cholamidopropyl)dimethylammonio]-1-propanesulfonate
CID: collision induced dissociation
CPP: cerato-platinin-related protein
GPI: glycosylphosphatidylinositol
HPLC: high-performance liquid chromatography
HR: hypersensitive response
ID: internal diameter
IPG: immobilized pH gradient
LC-MS/MS: liquid chromatography coupled to tandem mass spectrometry
MeSA: methylsalicylic acid
PCD: programmed cell death
ROS: reactive oxygen species
SA: salicylic acid
SABP: SA binding protein
SAR: systemic acquired resistance
SDS-PAGE: sodium dodecylsulfate polyacrylamide gel electrophoresis
SSA: 5-sulfosalicylic acid
TCA: trichloroacetic acid.

## References

[B1] Aebersold R. Mann M. (2003). Mass spectrometry-based proteomics. Nature 422, 198–207. 10.1038/nature01511 12634793

[B2] Aguileta G. Lengellé J. Chiapello H. Giraud T. Viaud M. Fournier E.. (2012). Genes under positive selection in a model plant pathogenic fungus. *Botrytis*. Infect. Genet. Evol. 12, 987–996. 10.1016/j.meegid.2012.02.012 22406010

[B3] Alem M. A. S. Douglas L. J. (2004). Effects of aspirin and other nonsteroidal anti-inflammatory drugs on biofilms and planktonic cells of *Candida albicans*. Antimicrob. Agents Chemother. 48, 41–47. 10.1128/AAC.48.1.41-47.2004 14693516 PMC310207

[B4] Ali B. Kaur S. (1983). Mammalian tissue acetylsalicylic acid esterase(s): identification, distribution and discrimination from other esterases. J. Pharmacol. Exp. Ther. 226, 589–594. 6875867

[B5] Amborabé B. E. Fleurat-Lessard P. Chollet J. F. Roblin G. (2002). Antifungal effects of salicylic acid and other benzoic acid derivatives towards *Eutypa lata*: structure–activity relationship. Plant Physiol. Biochem. 40, 1051–1060. 10.1016/s0981-9428(02)01470-5

[B6] Amich J. Leal F. Calera J. A. (2009). Repression of the acid ZrfA/ZrfB zinc-uptake system of *Aspergillus fumigatus* mediated by PacC under neutral, zinc-limiting conditions. Int. Microbiol. 12, 39–47. 10.2436/20.1501.01.80 19440982

[B7] Amselem J. Cuomo C. A. van Kan J. A. Viaud M. Benito E. P. Couloux A.. (2011). Genomic analysis of the necrotrophic fungal pathogens *Sclerotinia sclerotiorum* and *Botrytis cinerea*. PLoS Genet. 7:e1002230. 10.1371/journal.pgen.1002230 21876677 PMC3158057

[B8] An C. Mou Z. (2011). Salicylic acid and its function in plant immunity. J. Integr. Plant Biol. 53, 412–428. 10.1111/j.1744-7909.2011.01043.x 21535470

[B9] Ariño J. (2010). Integrative responses to high pH stress in *S. cerevisiae*. OMICS 14, 517–523. 10.1089/omi.2010.0044 20726779

[B10] Avenot H. F. Michailides T. J. (2010). Progress in understanding molecular mechanisms and evolution of resistance to succinate dehydrogenase inhibiting (SDHI) fungicides in phytopathogenic fungi. Crop Prot. 29, 643–651. 10.1016/j.cropro.2010.02.019

[B11] Baccelli I. (2015). Cerato-platanin family proteins: one function for multiple biological roles? Front. Plant Sci. 5:769. 10.3389/fpls.2014.00769 25610450 PMC4284994

[B12] Belenky P. Camacho D. Collins J. J. (2013). Fungicidal drugs induce a common oxidative-damage cellular death pathway. Cell Rep. 3, 350–358. 10.1016/j.celrep.2012.12.021 23416050 PMC3656588

[B13] Bendtsen J. D. Jensen L. J. Blom N. von Heijne G. Brunak S. (2004a). Feature based prediction of non-classical and leaderless protein secretion. Protein Eng. Des. Sel. 17, 349–356. 10.1093/protein/gzh037 15115854

[B14] Bendtsen J. D. Nielsen H. von Heijne G. Brunak S. (2004b). Improved prediction of signal peptides: signalP 3.0. J. Mol. Biol. 340, 783–795. 10.1016/j.jmb.2004.05.028 15223320

[B15] Bianco L. Perrotta G. (2015). Methodologies and perspectives of proteomics applied to filamentous fungi: from sample preparation to secretome analysis. Int. J. Mol. Sci. 16, 5803–5829. 10.3390/ijms16035803 25775160 PMC4394507

[B16] Bolton M. D. Thomma B. P. Nelson B. D. (2006). *Sclerotinia sclerotiorum* (Lib.) de Bary: biology and molecular traits of a cosmopolitan pathogen. Mol. Plant Pathol. 7, 1–16. 10.1111/j.1364-3703.2005.00316.x 20507424

[B17] Brown N. A. Antoniw J. Hammond-Kosack K. E. (2012). The predicted secretome of the plant pathogenic fungus *Fusarium graminearum*: A refined comparative analysis. PLoS ONE 7:e33731. 10.1371/journal.pone.0033731 22493673 PMC3320895

[B18] Caarls L. Pigeeterse C. M. Van Wees S. C. (2015). How salicylic acid takes transcriptional control over jasmonic acid signaling. Front. Plant Sci. 6:170. 10.3389/fpls.2015.00170 25859250 PMC4373269

[B19] Caccia D. Dugo M. Callari M. Bongarzone I. (2013). Bioinformatics tools for secretome analysis. Biochim. Biophys. Acta 1834, 2442–2453. 10.1016/j.bbapap.2013.01.039 23395702

[B20] Cameron R. K. Zaton K. (2004). Intercellular salicylic acid accumulation is important for age-related resistance in *Arabidopsis* to *Pseudomonas syringae*. Physiol. Mol. Plant P. 65, 197–209. 10.1016/j.pmpp.2005.02.002

[B21] Carviel J. L. Al-Daoud F. Neumann M. Mohammad A. Provart N. J. Moeder W.. (2009). Forward and reverse genetics to identify genes involved in the age-related resistance response in *Arabidopsis thaliana*. Mol. Plant Pathol. 10, 621–634. 10.1111/j.1364-3703.2009.00557.x 19694953 PMC6640485

[B22] Carviel J. L. Wilson D. C. Isaacs M. Carella P. Catana V. Golding B.. (2014). Investigation of intercellular salicylic acid accumulation during compatible and incompatible *Arabidopsis-Pseudomonas syringae* interactions using a fast neutron-generated mutant allele of *EDS5* identified by genetic mapping and whole-genome sequencing. PLoS ONE 9:e88608. 10.1371/journal.pone.0088608 24594657 PMC3942312

[B23] Catusse J. Strub J. M. Job C. Van Dorsselaer A. Job D. (2008). Proteome-wide characterization of sugarbeet seed vigor and its tissue specific expression. Proc. Natl. Acad. Sci. U.S.A. 29, 10262–10267. 10.1073/pnas.0800585105 18635686 PMC2474813

[B24] Chabane S. Sarfati J. Ibrahim-Granet O. Du C. Schmidt C. Mouyna I.. (2006). Glycosylphosphatidylinositol-anchored Ecm33p influences conidial cell wall biosynthesis in *Aspergillus fumigatus*. Appl. Environ. Microbiol. 72, 3259–3267. 10.1128/aem.72.5.3259-3267.2006 16672465 PMC1472355

[B25] Chen F. D'Auria J. C. Tholl D. Ross J. R. Gershenzon J. Noel J. P.. (2003). An *Arabidopsis thaliana* gene for methylsalicylate biosynthesis, identified by a biochemical genomics approach, has a role in defense. Plant J. 36, 577–588. 10.1046/j.1365-313X.2003.01902.x 14617060

[B26] Chen Z. X. Silva H. Klessig D. F. (1993). Active oxygen species in the induction of plant systemic acquired-resistance by salicylic acid. Science 262, 1883–1886. 10.1126/science.8266079 8266079

[B27] Cherrad S. Girard V. Dieryckx C. Gonçalves I. R. Dupuy J. W. Bonneu M.. (2012). Proteomic analysis of proteins secreted by *Botrytis cinerea* in response to heavy metal toxicity. Metallomics 4, 835–846. 10.1039/c2mt20041d 22706205

[B28] Chevallet M. Diemer H. Van Dorssealer A. Villiers C. Rabilloud T. (2007). Toward a better analysis of secreted proteins: the example of the myeloid cells secretome. Proteomics 7, 1757–1770. 10.1002/pmic.200601024 17464941 PMC2386146

[B29] Choi J. Park J. Kim D. Jung K. Kang S. Lee Y. H. (2010). Fungal secretome database: integrated platform for annotation of fungal secretomes. BMC Genomics 11:105. 10.1186/1471-2164-11-105 20146824 PMC2836287

[B30] Cobos R. Barreiro C. Mateos R. M. Coque J. J. R. (2010). Cytoplasmic- and extracellular-proteome analysis of *Diplodia seriata*: a phytopathogenic fungus involved in grapevine decline. Proteome Sci. 8:46. 10.1186/1477-5956-8-46 20828386 PMC2944164

[B31] Conde e Silva N. Gonçalves I. R. Lemaire M. Lesuisse E. Camadro J. M. Blaiseau P. L. (2009). KlAft, the *Kluyveromyces lactis* ortholog of Aft1 and Aft2, mediates activation of iron-responsive transcription through the PuCACCC Aft-type sequence. Genetics 183, 93–106. 10.1534/genetics.109.104364 19581449 PMC2746170

[B32] Cory A. H. Cory J. G. (2005). Phenolic compounds, sodium salicylate and related compounds, as inhibitors of tumor cell growth and inducers of apoptosis in mouse leukemia L1210 cells. In Vivo 19, 31–36. 15796154

[B33] Dangl J. L. Jones J. D. G. (2001). Plant pathogens and integrated defence responses to infection. Nature 411, 826–833. 10.1038/35081161 11459065

[B34] Dean R. Van Kan J. A. Pretorius Z. A. Hammond-Kosack K. E. Di Pietro A. Spanu P. D.. (2012). The top 10 fungal pathogens in molecular plant pathology. Mol. Plant Pathol. 13, 414–430. 10.1111/j.1364-3703.2011.00783.x 22471698 PMC6638784

[B35] Delaunois B. Jeandet P. Clement C. Baillieul F. Dorey S. Cordelier S. (2014). Uncovering plant-pathogen crosstalk through apoplastic proteomic studies. Front. Plant Sci. 5:249. 10.3389/fpls.2014.00249 24917874 PMC4042593

[B36] Dempsey D. A. Vlot A. C. Wildermuth M. C. Klessig D. F. (2011). Salicylic acid biosynthesis and metabolism. Arabidopsis Book 9:e0156. 10.1199/tab.0156 22303280 PMC3268552

[B37] de Oliveira A. L. Gallo M. Pazzagli L. Benedetti C. E. Cappugi G. Scala A.. (2011). The structure of the elicitor cerato-platanin (CP), the first member of the CP fungal protein family, reveals a double ψβ-barrel fold and carbohydrate binding. J. Biol. Chem. 286, 17560–17568. 10.1074/jbc.M111.223644 21454637 PMC3093830

[B38] Durner J. Klessig D. F. (1995). Inhibition of ascorbate peroxidase by salicylic acid and 2,6-dichloroisonicotinic acid, 2 inducers of plant defense responses. Proc. Natl. Acad. Sci. U.S.A. 92, 11312–11316. 10.1073/pnas.92.24.11312 7479986 PMC40622

[B39] Durrant W. E. Dong X. (2004). Systemic acquired resistance. Annu. Rev. Phytopathol. 42, 185–209. 10.1146/annurev.phyto.42.040803.140421 15283665

[B40] Eisendle M. Oberegger H. Buttinger R. Illmer P. Haas H. (2004). Biosynthesis and uptake of siderophores is controlled by the PacC-mediated ambient-pH regulatory system in *Aspergillus nidulans*. Eukaryot. Cell 3, 561–563. 10.1128/EC.3.2.561-563.2004 15075286 PMC387658

[B41] Elad Y. (1997). Responses of plants to infection by *Botrytis cinerea* and novel means involved in reducing their susceptibility to infection. Biol. Rev. 72, 381–422. 10.1017/S0006323197005057

[B42] El Oirdi M. El Rahman T. A. Rigano L. El Hadrami A. Rodriguez M. C. Daayf F.. (2011). *Botrytis cinerea* manipulates the antagonistic effects between immune pathways to promote disease development in tomato. Plant Cell 23, 2405–2421. 10.1105/tpc.111.083394 21665999 PMC3160041

[B43] Emanuelsson O. Brunak S. von Heijne G. Nielsen H. (2007). Locating proteins in the cell using TargetP, SignalP, and related tools. Nat. Protoc. 2, 953–971. 10.1038/nprot.2007.131 17446895

[B44] Erjavec J. Kos J. Ravnikar M. Dreo T. Sabotič J. (2012). Proteins of higher fungi: from forest to application. Trends Biotechnol. 30, 259–273. 10.1016/j.tibtech.2012.01.004 22341093

[B45] Espino J. J. Gutiérrez-Sánchez G. Brito N. Shah P. Orlando R. González C. (2010). The *Botrytis cinerea* early secretome. Proteomics 10, 3020–3034. 10.1002/pmic.201000037 20564262 PMC3983782

[B46] Fernandes I. Alves A. Correia A. Devreese B. Esteves A. C. (2014). Secretome analysis identifies potential virulence factors of *Diplodia corticola*, a fungal pathogen involved in cork oak (*Quercus suber*) decline. Fungal Biol. 18, 516–523. 10.1016/j.funbio.2014.04.006 24863480

[B47] Fernández-Acero F. J. Colby T. Harzen A. Carbú M. Wieneke U. Cantoral J. M.. (2010). 2-DE proteomic approach to the *Botrytis cinerea* secretome induced with different carbon sources and plant-based elicitors. Proteomics 10, 2270–2280. 10.1002/pmic.200900408 20376862

[B48] Fernández-Acero F. J. Jorge I. Calvo E. Vallejo I. Carbú M. Camafeita E.. (2006). Two-dimensional electrophoresis protein profile of the phytopathogenic fungus *Botrytis cinerea*. Proteomics 6, S88–S96. 10.1002/pmic.200500436 16544282

[B49] Ferrari S. Plotnikova J. M. De Lorenzo G. Ausubel F. M. (2003). *Arabidopsis* local resistance to *Botrytis cinerea* involves salicylic acid and camalexin and requires *EDS4* and *PAD2*, but not *SID2, EDS5* or *PAD4*. Plant J. 35, 193–205. 10.1046/j.1365-313X.2003.01794.x 12848825

[B50] Forouhar F. Yang Y. Kumar D. Chen Y. Fridman E. Park S. W.. (2005). Structural and biochemical studies identify tobacco SABP2 as a methyl salicylate esterase and implicate it in plant innate immunity. Proc. Natl. Acad. Sci. U.S.A. 102, 1773–1778. 10.1073/pnas.0409227102 15668381 PMC547883

[B51] Frías M. Brito N. González C. (2013). The *Botrytis cinerea* cerato-platanin BcSpl1 is a potent inducer of systemic acquired resistance (SAR) in tobacco and generates a wave of salicylic acid expanding from the site of application. Mol. Plant Pathol. 14, 191–196. 10.1111/j.1364-3703.2012.00842.x 23072280 PMC6638659

[B52] Frías M. Brito N. González M. González C. (2014). The phytotoxic activity of the cerato-platanin BcSpl1 resides in a two-peptide motif on the protein surface. Mol. Plant Pathol. 15, 342–351. 10.1111/mpp.12097 24175916 PMC6638778

[B53] Frías M. González C. Brito N. (2011). BcSpl1, a cerato-platanin family protein, contributes to *Botrytis cinerea* virulence and elicits the hypersensitive response in the host. New Phytol. 192, 483–495. 10.1111/j.1469-8137.2011.03802.x 21707620

[B54] Gaderer R. Bonazza K. Seidl-Seiboth V. (2014). Cerato-platanins: a fungal protein family with intriguing properties and application potential. Appl. Microbiol. Biotechnol. 98, 4795–4803. 10.1007/s00253-014-5690-y 24687753 PMC4024134

[B55] Gaffney T. Friedrich L. Vernooij B. Negrotto D. Nye G. Uknes S.. (1993). Requirement of salicylic acid for the induction of systemic acquired resistance. Science 261, 754–756. 10.1126/science.261.5122.754 17757215

[B56] Gamborg O. L. Miller R. A. Ojima K. (1968). Nutrient requirements of suspension cultures of soybean root cells. Exp. Cell Res. 50, 151–158. 10.1016/0014-4827(68)90403-5 5650857

[B57] Gisi U. Sierotzki H. Cook A. McCaffery A. (2002). Mechanisms influencing the evolution of resistance to Qo inhibitor fungicides. Pest Manag. Sci. 58, 859–867. 10.1002/ps.565 12233175

[B58] Gómez-Mendoza D. P. Junqueira M. do Vale L. H. Domont G. B. Ferreira Filho E. X. Sousa M. V.. (2014). Secretomic survey of *Trichoderma harzianum* grown on plant biomass substrates. J. Proteome Res. 13, 1810–1822. 10.1021/pr400971e 24593137

[B59] González M. Brito N. Frías M. González C. (2013). *Botrytis cinerea* protein *O*-mannosyltransferases play critical roles in morphogenesis, growth, and virulence. PLoS ONE 8, e65924. 10.1371/journal.pone.0065924 23762450 PMC3675079

[B60] González M. Brito N. González C. (2014). Identification of glycoproteins secreted by wild-type *Botrytis cinerea* and by protein *O*-mannosyltransferase mutants. BMC Microbiol. 14:254. 10.1186/s12866-014-0254-y 25305780 PMC4197228

[B61] González-Fernández R. Aloria K. Valero-Galván J. Redondo I. Arizmendi J. M. Jorrín-Novo J. V. (2014). Proteomic analysis of mycelium and secretome of different *Botrytis cinerea* wild-type strains. J. Proteomics 97, 195–221. 10.1016/j.jprot.2013.06.022 23811051

[B62] González-Fernández R. Jorrín-Novo J. V. (2012). Contribution of proteomics to the study of plant pathogenic fungi. J. Proteome Res. 11, 3–16. 10.1021/pr200873p 22085090

[B63] Hahn M. Viaud M. van Kan J. (2014). The genome of Botrytis cinerea, a ubiquitous broad host range necrotroph, in Genomics of Plant-associated Fungi and Oomycetes: Dicot Pathogens, eds Dean R. A. Lichens-Park A. Kole C. (Berlin; Heidelberg: Springer-Verlag), 19–44.

[B64] Hayat Q. Hayat S. Irfan M. Ahmad A. (2010). Effect of exogenous salicylic acid under changing environment: a review. Environ. Exp. Bot. 68, 14–25. 10.1016/j.envexpbot.2009.08.005

[B65] Heard S. Brown N. A. Hammond-Kosack K. (2015). An interspecies comparative analysis of the predicted secretomes of the necrotrophic plant pathogens *Sclerotinia sclerotiorum* and *Botrytis cinerea*. PloS ONE 10:e0130534. 10.1371/journal.pone.0130534 26107498 PMC4480369

[B66] Huang W. E. Huang L. Preston G. M. Naylor M. Carr J. P. Li Y.. (2006). Quantitative *in situ* assay of salicylic acid in tobacco leaves using a genetically modified biosensor strain of *Acinetobacter* sp. ADP1. Plant J. 46, 1073–1083. 10.1111/j.1365-313X.2006.02758.x 16805738

[B67] Jashni M. K. Mehrabi R. Collemare J. Mesarich C. H. de Wit P. J. (2015). The battle in the apoplast: further insights into the roles of proteases and their inhibitors in plant–pathogen interactions. Front. Plant Sci. 6:584. 10.3389/fpls.2015.00584 26284100 PMC4522555

[B68] Jones A. W. (2011). Early drug discovery and the rise of pharmaceutical chemistry. Drug Test. Analysis 3, 337–344. 10.1002/dta.301 21698778

[B69] Jung Y. H. Jeong S. H. Kim S. H. Singh R. Lee J. E. Cho Y. S.. (2012). Secretome analysis of *Magnaporthe oryzae* using *in vitro* systems. Proteomics 12, 878–900. 10.1002/pmic.201100142 22539438

[B70] Kim D. H. Yang Y. S. Jakoby W. B. (1990). Aspirin hydrolyzing esterases from rat liver cytosol. Biochem. Pharmacol. 40, 481–487. 10.1016/0006-2952(90)90546-w 2383281

[B71] Krupka L. R. Racle F. A. Marderosian A. D. (1967). Degradation of salicylate by *Aspergillus niger*. Nature 216, 486–487. 10.1038/216486a0 6057252

[B72] Kumar D. Klessig D. F. (2003). High-affinity salicylic acid-binding protein 2 is required for plant innate immunity and has salicylic acid-stimulated lipase activity. Proc. Natl. Acad. Sci. U.S.A. 100, 16101–16106. 10.1073/pnas.0307162100 14673096 PMC307699

[B73] Laemmli U. K. (1970). Cleavage of structural proteins during the assembly of the head of bacteriophage T4. Nature 227, 680–685. 10.1038/227680a0 5432063

[B74] Lamdan N. L. Shalaby S. Ziv T. Kenerley C. M. Horwitz B. A. (2015). Secretome of *Trichoderma* interacting with maize roots: role in induced systemic resistance. Mol. Cell. Proteomics 14, 1054–1063. 10.1074/mcp.m114.046607 25681119 PMC4390251

[B75] Lebeis S. L. Paredes S. H. Lundberg D. S. Breakfield N. Gehring J. McDonald M.. (2015). Salicylic acid modulates colonization of the root microbiome by specific bacterial taxa. Science 349, 860–864. 10.1126/science.aaa8764 26184915

[B76] Leeuw N. J. Swart C. W. Ncango D. M. Kriel W. M. Pohl C. H. van Wyk P. W. J.. (2009). Anti-inflammatory drugs selectively target sporangium development in Mucor. Can. J. Microbiol. 55, 1392–1396. 10.1139/w09-096 20029531

[B77] Leeuw N. J. Swart C. W. Ncango D. M. Pohl C. H. Sebolai O. M. Strauss C. J.. (2007). Acetylsalicylic acid as antifungal in *Eremothecium* and other yeasts. Anton. Leeuw. Int. J. G. 91, 393–405. 10.1007/s10482-006-9124-4 17094014

[B78] Lemos M. F. Soares A. M. Correia A. C. Esteves A. C. (2010). Proteins in ecotoxicology - how, why and why not? Proteomics 10, 873–887. 10.1002/pmic.200900470 19953548

[B79] Leo A. Hansch C. ElkIns I. (1971). Partition coefficients and their uses. Chem. Rev. 71, 525–616. 10.1021/cr60274a001

[B80] Li B. Wang W. Zong Y. Qin G. Tian S. (2012). Exploring pathogenic mechanisms of *Botrytis cinerea* secretome under different ambient pH based on comparative proteomic analysis. J. Proteome Res. 11, 4249–4260. 10.1021/pr300365f 22746291

[B81] Li S. C. Kane P. M. (2009). The yeast lysosome-like vacuole: endpoint and crossroads. BBA-Mol. Cell Res. 1793, 650–663. 10.1016/j.bbamcr.2008.08.003 18786576 PMC2906225

[B82] Linde J. Wilson D. Hube B. Guthke R. (2010). Regulatory network modelling of iron acquisition by a fungal pathogen in contact with epithelial cells. BMC Syst. Biol. 4:148. 10.1186/1752-0509-4-148 21050438 PMC3225834

[B83] Liu T. Song T. Zhang X. Yuan H. Su L. Li W.. (2014). Unconventionally secreted effectors of two filamentous pathogens target plant salicylate biosynthesis. Nat. Comm. 5, 4686. 10.1038/ncomms5686 25156390 PMC4348438

[B84] Lu X. Sun J. Nimtz M. Wissing J. Zeng A. P. Rinas U. (2010). The intra- and extracellular proteome of *Aspergillus niger* growing on defined medium with xylose or maltose as carbon substrate. Microb. Cell Fact. 9:23. 10.1186/1475-2859-9-23 20406453 PMC2874515

[B85] Mansfield J. W. (1980). Mechanisms of resistance to *Botrytis*, in The Biology of Botrytis, eds Coley-Smith J. R. Verhoeff K. Jarvis W. R. (London: Academic Press), 81–218.

[B86] Martinez-Lopez R. Park H. Myers C. L. Gil C. Filler S. G. (2006). *Candida albicans* Ecm33p is important for normal cell wall architecture and interactions with host cells. Eukaryot. Cell 5, 140–147. 10.1128/EC.5.1.140-147.2006 16400176 PMC1360258

[B87] Medina M. J. H. Gagnon H. Piché Y. Ocampo J. A. Garrido J. M. G. Vierheilig H. (2003). Root colonization by arbuscular mycorrhizal fungi is affected by the salicylic acid content of the plant. Plant Sci. 164, 993–998. 10.1016/S0168-9452(03)00083-9

[B88] Medina M. L. Haynes P. A. Breci L. Francisco W. A. (2005). Analysis of secreted proteins from *Aspergillus flavus*. Proteomics 5, 3153–3161. 10.1002/pmic.200401136 16035112

[B89] Mei X. Yang M. Ding X. Bi Y. Chen L. Deng W.. (2014). Proteomic analysis of zoxamide-induced changes in *Phytophthora cactorum*. Pestic. Biochem. Phys. 113, 31–39. 10.1016/j.pestbp.2014.06.004 25052524

[B90] Mengiste T. (2012). Plant immunity to necrotrophs. Annu. Rev. Phytopathol. 50, 267–294. 10.1146/annurev-phyto-081211-172955 22726121

[B91] Meyer M. C. Bueno C. J. de Souza N. L. Yorinori J. T. (2006). Effect of doses of fungicides and plant resistance activators on the control of Rhizoctonia foliar blight of soybean, and on *Rhizoctonia solani* AG1-IA *in vitro* development. Crop Prot. 25, 848–854. 10.1016/j.cropro.2005.11.008

[B92] Moret A. Nadal M. Munoz Z. (2007). Assay of the fungicidal action of acetylsalicylic acid on *Botrytis cinerea*. Acta Hortic. 754, 367–371. 10.17660/actahortic.2007.754.48

[B93] Naglik J. R. Challacombe S. J. Hube B. (2003). *Candida albicans* secreted aspartyl proteinases in virulence and pathogenesis. Microbiol. Mol. Biol. R. 67, 400–428. 10.1128/mmbr.67.3.400-428.2003 12966142 PMC193873

[B94] Nickel W. (2003). The mystery of nonclassical protein secretion - A current view on cargo proteins and potential export routes. Eur. J. Biochem. 270, 2109–2119. 10.1046/j.1432-1033.2003.03577.x 12752430

[B95] Nulton-Persson A. C. Szweda L. I. Sadek H. A. (2004). Inhibition of cardiac mitochondrial respiration by salicylic acid and acetylsalicylate. J. Cardiovasc. Pharmacol. 44, 591–595. 10.1097/00005344-200411000-00012 15505497

[B96] Oda K. Kakizono D. Yamada O. Iefuji H. Akita O. Iwashita K. (2006). Proteomic analysis of extracellular proteins from *Aspergillus oryzae* grown under submerged and solid-state culture conditions. Appl. Environ. Microbiol. 72, 3448–3457. 10.1128/aem.72.5.3448-3457.2006 16672490 PMC1472361

[B97] Panahirad S. Zaare-Nahandi F. Mohammadi N. Alizadeh-Salteh S. Safaie N. (2014). Effects of salicylic acid on *Aspergillus flavus* infection and aflatoxin B1 accumulation in pistachio (*Pistacia vera* L.) fruit. J. Sci. Food Agric. 94, 1758–1763. 10.1002/jsfa.6488 24272956

[B98] Pardo M. Monteoliva L. Vázquez P. Martínez R. Molero G. Nombela C.. (2004). *PST1* and *ECM33* encode two yeast cell surface GPI proteins important for cell wall integrity. Microbiology 150, 4157–4170. 10.1099/mic.0.26924-0 15583168

[B99] Park S. W. Kaimoyo E. Kumar D. Mosher S. Klessig D. F. (2007). Methyl salicylate is a critical mobile signal for plant systemic acquired resistance. Science 318, 113–116. 10.1126/science.1147113 17916738

[B100] Pazzagli L. Cappugi G. Manao G. Camici G. Santini A. Scala A. (1999). Purification, characterization, and amino acid sequence of cerato-platanin, a new phytotoxic protein from *Ceratocystis fimbriata* f. sp. *platani*. J. Biol. Chem. 274, 24959–24964. 10.1074/jbc.274.35.24959 10455173

[B101] Peñalva M. A. Tilburn J. Bignell E. Arst H. N. Jr. (2008). Ambient pH gene regulation in fungi: making connections. Trends Microbiol. 16, 291–300. 10.1016/j.tim.2008.03.006 18457952

[B102] Penn C. D. Daniel S. L. (2013). Salicylate degradation by the fungal plant pathogen *Sclerotinia sclerotiorum*. Curr. Microbiol. 67, 218–225. 10.1007/s00284-013-0349-y 23512122

[B103] Perrin D. D. (1958). Stability of metal complexes with salicylic acid and related substances. Nature 182, 741–742. 10.1038/182741a0 13590098

[B104] Prithiviraj B. Manickam M. Singh U. P. Ray A. B. (1997). Antifungal activity of anacardic acid, a naturally occurring derivative of salicylic acid. Can. J. Bot. 75, 207–211. 10.1139/b97-021

[B105] Qi P. F. Johnston A. Balcerzak M. Rocheleau H. Harris L. J. Long X. Y.. (2012). Effect of salicylic acid on *Fusarium graminearum*, the major causal agent of Fusarium head blight in wheat. Fungal Biol. 116, 413–426. 10.1016/j.funbio.2012.01.001 22385623

[B106] Rabe F. Ajami-Rashidi Z. Doehlemann G. Kahmann R. Djamei A. (2013). Degradation of the plant defence hormone salicylic acid by the biotrophic fungus *Ustilago maydis*. Mol. Microbiol. 89, 179–188. 10.1111/mmi.12269 23692401

[B107] Rajjou L. Belghazi M. Huguet R. Robin C. Moreau A. Job C.. (2006). Proteomic investigation of the effect of salicylic acid on Arabidopsis seed germination and establishment of early defense mechanisms. Plant Physiol. 141, 910–923. 10.1104/pp.106.082057 16679420 PMC1489900

[B108] Rivas-San Vicente M. Plasencia J. (2011). Salicylic acid beyond defence: its role in plant growth and development. J. Exp. Bot. 62, 3321–3338. 10.1093/jxb/err031 21357767

[B109] Rolland S. Bruel C. Rascle C. Girard V. Billon-Grand G. Poussereau N. (2009). pH controls both transcription and post- translational processing of the protease BcACP1 in the phytopathogenic fungus *Botrytis cinerea*. Microbiology 155, 2097–2105. 10.1099/mic.0.025999-0 19359322

[B110] Ross J. R. Nam K. H. D'Auria J. C. Pichersky E. (1999). *S*-adenosyl-L-methionine:salicylic acid carboxyl methyltransferase, an enzyme involved in floral scent production and plant defense, represents a new class of plant methyltransferases. Arch. Biochem. Biophys. 367, 9–16. 10.1006/abbi.1999.1255 10375393

[B111] Rovenich H. Boshoven J. C. Thomma B. P. H. J. (2014). Filamentous pathogen effector functions: of pathogens, hosts and microbiomes. Curr. Opin. Plant Biol. 20, 96–103. 10.1016/j.pbi.2014.05.001 24879450

[B112] Schadler D. L. George A. A. (2006). Synthesis and bioassay of a volatile fungistatic agent. Plant Health Instr. 10.1094/PHI-I-2006-0717-02 Available online at: http://www.apsnet.org/edcenter/intropp/LabExercises/Pages/FungistaticAgent.aspx

[B113] Schwanhäusser B. Busse D. Li N. Dittmar G. Schuchhardt J. Wolf J.. (2011). Global quantification of mammalian gene expression control. Nature 473, 337–342. 10.1038/nature10098 21593866

[B114] Sebolai O. M. Pohl C. H. Botes P. J. van Wyk P. W. J. Mzizi R. Swart C. W.. (2008). Distribution of 3-hydroxy oxylipins and acetylsalicylic acid sensitivity in *Cryptococcus* species. Can. J. Microbiol. 54, 111–118. 10.1139/W07-116 18388980

[B115] Shah P. Atwood J. A. Orlando R. El Mubarek H. Podila G. K. Davis M. R. (2009). Comparative proteomic analysis of *Botrytis cinerea* secretome. J. Proteome Res. 8, 1123–1130. 10.1021/pr8003002 19140674

[B116] Shulaev V. Silverman P. Raskin I. (1997). Airborne signalling by methyl salicylate in plant pathogen resistance. Nature 385, 718–721. 10.1038/385718a0

[B117] Sierotzki H. Scalliet G. (2013). A review of current knowledge of resistance aspects for the next-generation succinate dehydrogenase inhibitor fungicides. Phytopathology 103, 880–887. 10.1094/PHYTO-01-13-0009-RVW 23593940

[B118] Spenney J. G. Nowell R. M. (1979). Acetylsalicylate hydrolase of rabbit gastric mucosa. Isolation and purification. Drug Metab. Dispos. 7, 215–259. 39723

[B119] Staats M. van Kan J. A. (2012). Genome update of *Botrytis cinerea* strains B05.10 and T4. Eukaryot. Cell 11, 1413–1414. 10.1128/EC.00164-12 23104368 PMC3486023

[B120] Stacey G. McAlvin C. B. Kim S. Y. Olivares J. Soto M. J. (2006). Effects of endogenous salicylic acid on nodulation in the model legumes *Lotus japonicus* and *Medicago truncatula*. Plant Physiol. 141, 1473–1481. 10.1104/pp.106.080986 16798946 PMC1533935

[B121] Stepanović S. Vuković D. Ješić M. Ranin L. (2004). Influence of acetylsalicylic acid (aspirin) on biofilm production by Candida species. J. Chemother. 16, 134–138. 10.1179/joc.2004.16.2.134 15216946

[B122] Swart C. W. van Wyk P. W. J. Pohl C. H. Kriel W. M. Kock J. L. F. (2011). The influence of mitochondrial inhibitors on the life cycle of *Phytophthora*. Afr. J. Microbiol. Res. 5, 3175–3180. 10.5897/AJMR11.139

[B123] ten Have A. Espino J. J. Dekkers E. Van Sluyter S. C. Brito N. Kay J.. (2010). *The Botrytis cinerea* aspartic proteinase family. Fungal Genet. Biol. 47, 53–65. 10.1016/j.fgb.2009.10.008 19853057

[B124] Tietjen K. Drewes M. Stenzel K. (2005). High throughput screening in agrochemical research. Comb. Chem. High Throughput Screen. 8, 589–594. 10.2174/138620705774575300 16305356

[B125] Tripathi D. Jiang Y. L. Kumar D. (2010). SABP2, a methyl salicylate esterase is required for the systemic acquired resistance induced by acibenzolar-*S*-methyl in plants. FEBS Lett. 584, 3458–3463. 10.1016/j.febslet.2010.06.046 20621100

[B126] Trofa D. Agovino M. Stehr F. Schäfer W. Rykunov D. Fiser A.. (2009). Acetylsalicylic acid (aspirin) reduces damage to reconstituted human tissues infected with Candida species by inhibiting extracellular fungal lipases. Microb. Infect. 11, 1131–1139. 10.1016/j.micinf.2009.08.007 19703582 PMC2787780

[B127] Unger S. H. Cook J. R. Hollenberg J. S. (1978). Simple procedure for determining octanol aqueous partition, distribution, and ionization coefficients by reversed-phase high-pressure liquid-chromatography. J. Pharm. Sci. 67, 1364–1367. 10.1002/jps.2600671008 702280

[B128] van Baarlen P. Woltering E. J. Staats M. van Kan J. A. L. (2007). Histochemical and genetic analysis of host and non-host interactions of *Arabidopsis* with three *Botrytis* species: an important role for cell death control. Mol. Plant Pathol. 8, 41–54. 10.1111/j.1364-3703.2006.00367.x 20507477

[B129] Vane J. R. Botting R. M. (2003). The mechanism of action of aspirin. Thromb. Res. 110, 255–258. 10.1016/S0049-3848(03)00379-7 14592543

[B130] van Loon L. C. Rep M. Pieterse C. M. J. (2006). Significance of inducible defense-related proteins in infected plants. Annu. Rev. Phytopathol. 44, 135–162. 10.1146/annurev.phyto.44.070505.143425 16602946

[B131] Vizcaíno J. A. Deutsch E. W. Wang R. Csordas A. Reisinger F. Ríos D.. (2014). ProteomeXchange provides globally coordinated proteomics data submission and dissemination. Nat. Biotechnol. 32, 223–226. 10.1038/nbt.2839 24727771 PMC3986813

[B132] Vlot A. C. Dempsey D. A. Klessig D. F. (2009). Salicylic acid, a multifaceted hormone to combat disease. Annu. Rev. Phytopathol. 47, 177–206. 10.1146/annurev.phyto.050908.135202 19400653

[B133] White K. N. Hope D. B. (1984). Partial purification and characterization of a microsomal carboxylesterase specific for salicylate esters from guinea-pig liver. Biochim. Biophys. Acta 785, 138–147. 10.1016/0167-4838(84)90138-9 6704404

[B134] Williamson B. Tudzynski B. Tudzynski P. van Kan J. A. L. (2007). *Botrytis ciner*ea: the cause of grey mould disease. Mol. Plant Pathol. 8, 561–580. 10.1111/j.1364-3703.2007.00417.x 20507522

[B135] Wu H. S. Raza W. Fan J. Q. Sun Y. G. Bao W. Liu D. Y.. (2008). Antibiotic effect of exogenously applied salicylic acid on *in vitro* soilborne pathogen, *Fusarium oxysporu*m f. sp. *niveum*. Chemosphere 74, 45–50. 10.1016/j.chemosphere.2008.09.027 18952255

[B136] Yang F. Jensen J. D. Svensson B. Jørgensen H. J. L. Collinge D. B. Finnie C. (2012). Secretomics identifies *Fusarium graminearum* proteins involved in the interaction with barley and wheat. Mol. Plant Pathol. 13, 445–453. 10.1111/j.1364-3703.2011.00759.x 22044785 PMC6638632

[B137] Yang Y. Zhang H. Li G. Li W. Wang X. Song F. (2009). Ectopic expression of MgSM1, a Cerato-platanin family protein from *Magnaporthe grisea*, confers broad-spectrum disease resistance in Arabidopsis. Plant Biotechnol. J. 7, 763–777. 10.1111/j.1467-7652.2009.00442.x 19754836

[B138] Yike I. (2011). Fungal proteases and their pathophysiological effects. Mycopathologia 171, 299–323. 10.1007/s11046-010-9386-2 21259054

[B139] Zhou Y. Wang G. Li Y. Liu Y. Song Y. Zheng W.. (2012). *In vitro* Interactions between Aspirin and Amphotericin B against planktonic cells and biofilm cells of *Candida albicans* and *C. parap*silosis. Antimicrob. Agents Chemother. 56, 3250–3260. 10.1128/aac.06082-11 22391539 PMC3370722

[B140] Zipfel C. (2009). Early molecular events in PAMP-triggered immunity. Curr. Opin. Plant Biol. 12, 414–420. 10.1016/j.pbi.2009.06.003 19608450

