# Supplementary figures and images for: Beyond plant defense: insights on the potential of salicylic and methylsalicylic acid to contain growth of the phytopathogen Botrytis cinerea
Source: Front Plant Sci. 2015 Oct 16;6:859. doi: 10.3389/fpls.2015.00859 (PMC4607878; doi:10.3389/fpls.2015.00859)

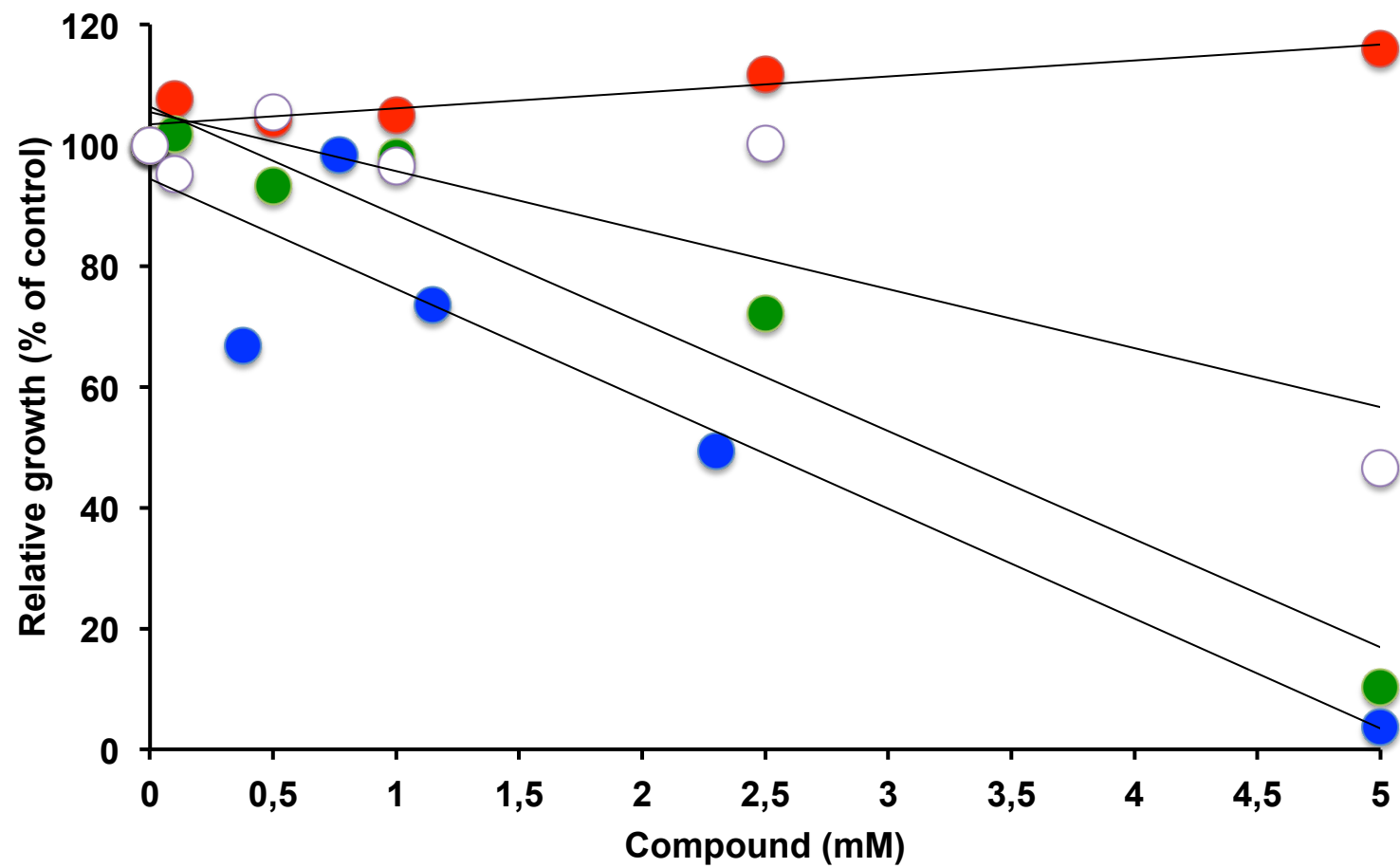

Supplement: Supplemental Figure S1 — Relative growth of Botrytis in the absence (control) or presence of the indicated compounds (ASA, SA, MeSA, and SSA). Measurements of mycelium growth (four replicates) were effected at 3 d of the cultures as detailed in Materials and Methods. [file Presentation1.PDF]

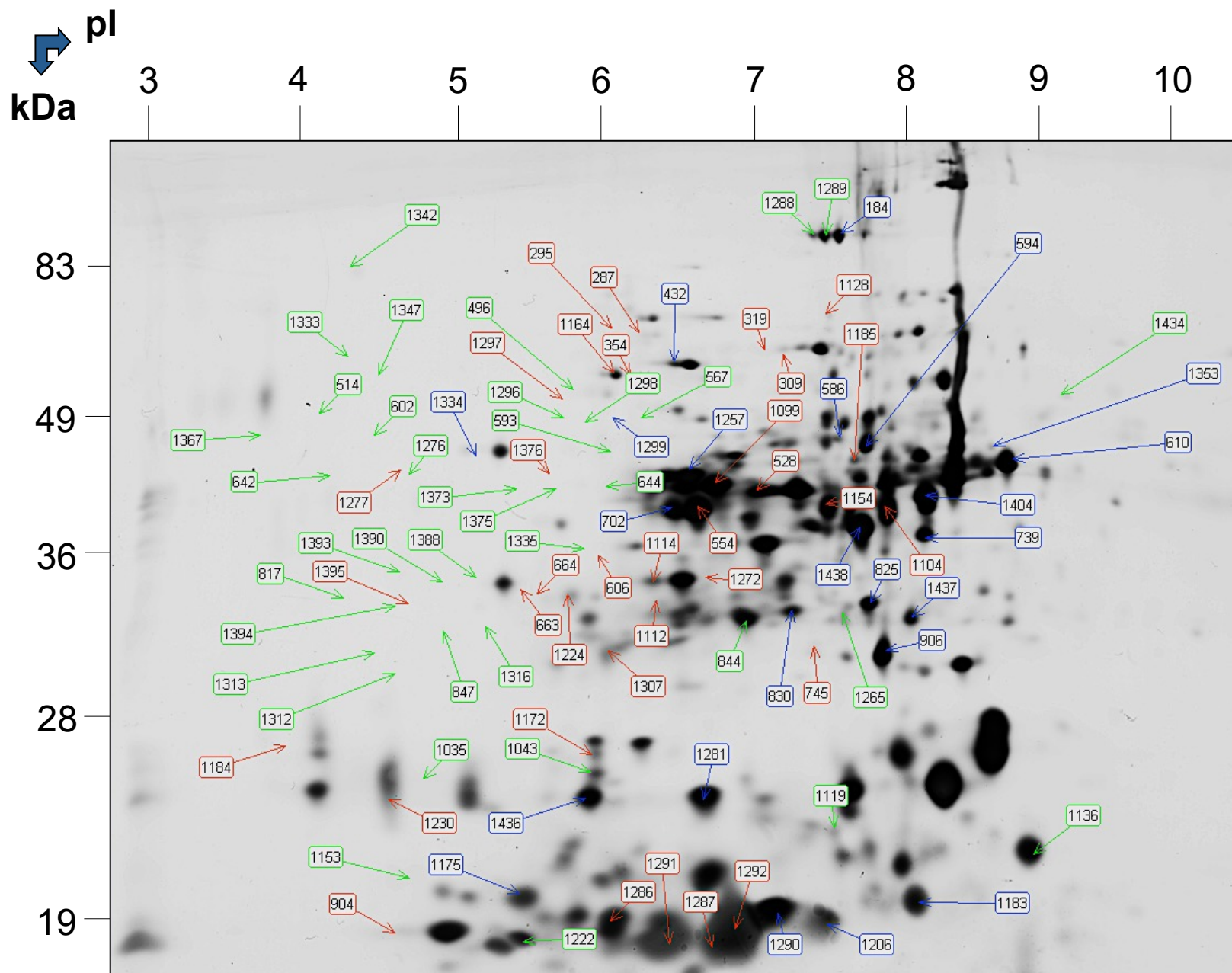

Supplement: Supplemental Figure S3 — Spot numbering for spots showing variations in spot volumes in the Botrytis intracellular mycelium proteome treated with MeSA or SA. The differentially accumulated spots are depicted on a typical 2D-gel corresponding to the intracellular proteome of untreated Botrytis control: Red arrows, differentially accumulated spots from MeSA-treated mycelium; green arrows, differentially accumulated spots from SA-treated mycelium; blue arrows, differentially accumulated spots from both MeSA- and SA-treated mycelium. The proteins contained in the various differentially accumulated spots are listed in Supplemental Table S1. [file Presentation3.PDF]

↙ pl  
kDa

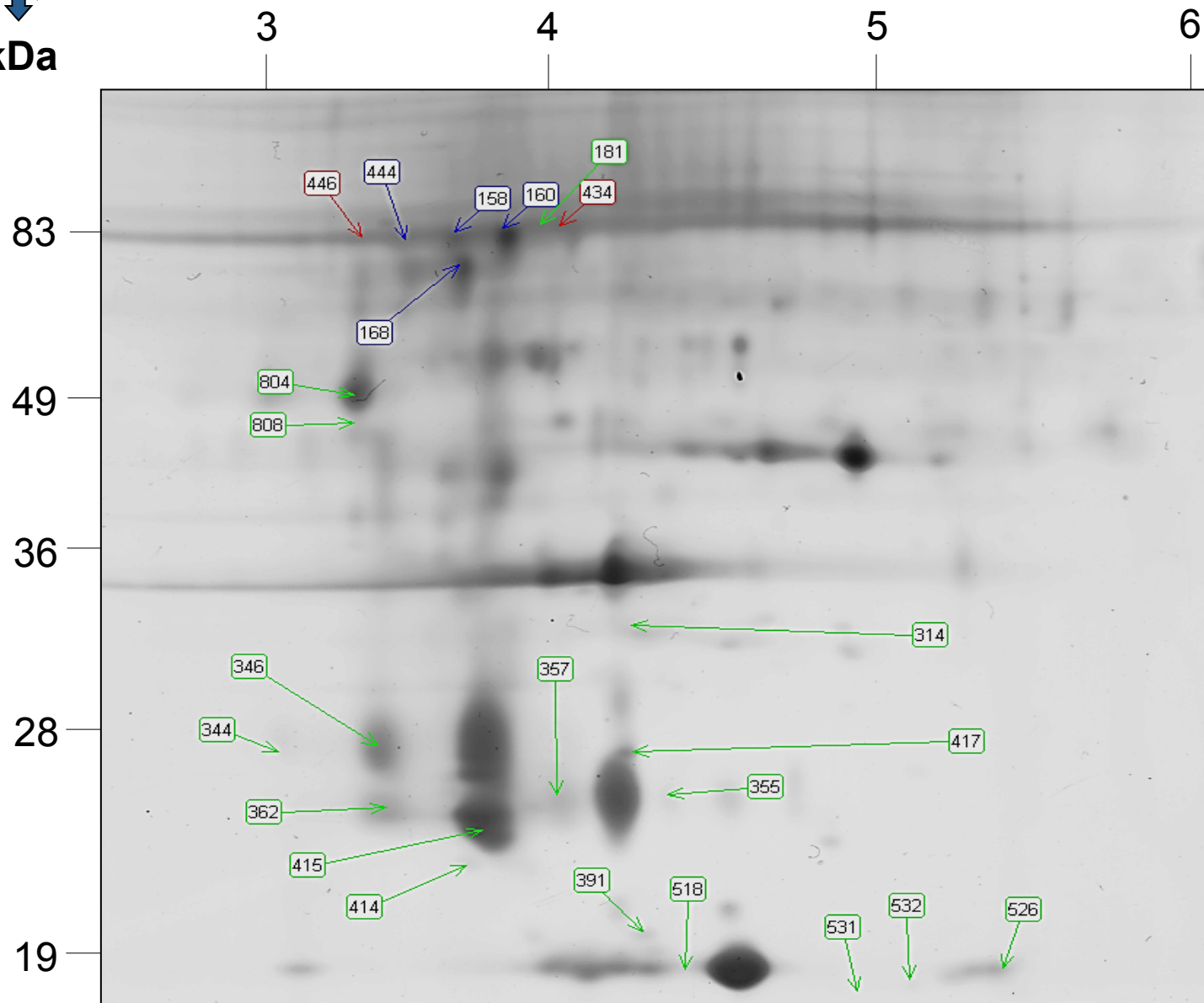

Supplement: Supplemental Figure S4 — Spot numbering for spots showing variations in spot volumes in the Botrytis extracellular mycelium secretome upon mycelium treatment with MeSA (0.38 mM) or SA (2.5 mM). The differentially accumulated spots are displayed on a typical 2D-gel corresponding to the extracellular secretome of control untreated Botrytis: Red arrows, differentially accumulated spots from MeSA-treated mycelium; green arrows, differentially accumulated spots from SA-treated mycelium; blue arrows, differentially accumulated spots from both MeSA- and SA-treated mycelium. The proteins contained in the various differentially accumulated spots are listed in Supplemental Table S2. [file Presentation4.PDF]
