# Supplementary material for: Beyond plant defense: insights on the potential of salicylic and methylsalicylic acid to contain growth of the phytopathogen Botrytis cinerea
Source: Front Plant Sci. 2015 Oct 16;6:859. doi: 10.3389/fpls.2015.00859 (PMC4607878; doi:10.3389/fpls.2015.00859)
Supplement: Supplemental Figure S2 — Distribution of the total absorbance in acidic (1) and basic (2) regions of 2D gels for intracellular mycelium proteome. (A) Acidic and basic regions of 2D gels. Gels shown corresponded to untreated mycelium grown at pH 5.0 (Control pH 5.0) or at pH 7.0 (Control pH 7.0) or to MeSA-treated mycelium grown at pH 5.0 (MeSA pH 5.0) or to SA-treated mycelium grown at pH 5.0 (SA pH 5.0). The acidic region (1) corresponded to the 3.0–6.0 pI range and the basic region corresponded to the 6.0–10.0 pI range of the 2D gels (separated by the blue lines). (B) Measurements of total absorbance in regions 1 and 2 of the 2D gels shown in (A). The total absorbance in each of the two regions was measured using the software Mesurim (http://acces.ens-lyon.fr/acces/logiciels/mesurim/guide-dutilisation/mesures-sur-limage#lumsurf) and three replicates for each condition. The ratios of total absorbance in region 2/total absorbance in region 1 are listed. [file Presentation2.PDF]

A

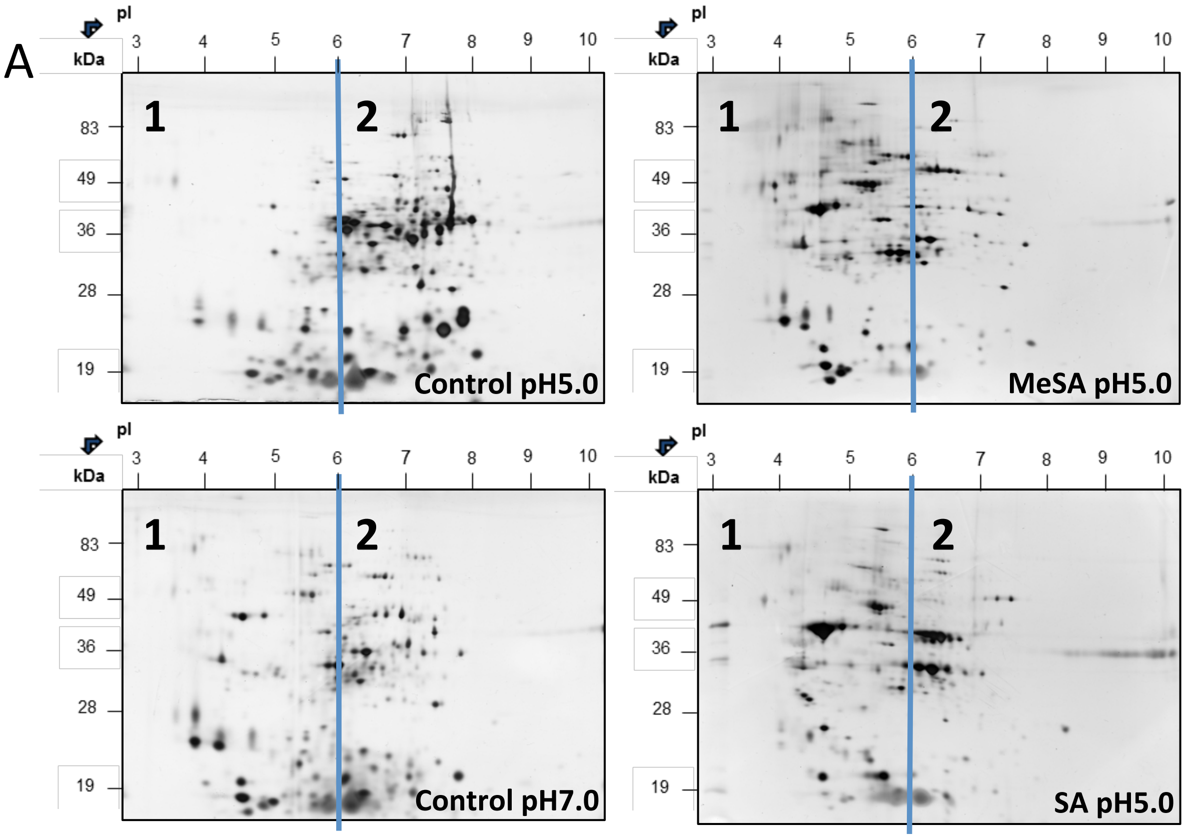

B

Gel condition	Average Absorbance Acid Region (1)	Average Absorbance Basic Region (2)	Absorbance ratio Basic (2)/Acid (1)
Control pH5.0	7.32	13.97	1.91
Control pH7.0	9.02	8.96	0.99
SA-treated pH5.0	14.67	11.89	0.81
MeSA-treated pH5.0	13.47	11.11	0.82
